# Acquired chemoresistance drives spatial heterogeneity, chemoprotection and collective migration in pancreatic tumor spheroids

**DOI:** 10.1371/journal.pone.0267882

**Published:** 2022-05-26

**Authors:** Fredrik I. Thege, Ian I. Cardle, Conor N. Gruber, Megan J. Siemann, Sophie Cong, Katharina Wittmann, Justin Love, Brian J. Kirby

**Affiliations:** 1 Cornell University, Ithaca, New York, United States of America; 2 MD Anderson Cancer Center, Houston, Texas, United States of America; 3 Weill Cornell Medicine, New York, New York, United States of America; Lobachevsky University, RUSSIAN FEDERATION

## Abstract

Tumors display rich cellular heterogeneity and typically consist of multiple co-existing clones with distinct genotypic and phenotypic characteristics. The acquisition of resistance to chemotherapy has been shown to contribute to the development of aggressive cancer traits, such as increased migration, invasion and stemness. It has been hypothesized that collective cellular behavior and cooperation of cancer cell populations may directly contribute to disease progression and lack of response to treatment. Here we show that the spontaneous emergence of chemoresistance in a cancer cell population exposed to the selective pressure of a chemotherapeutic agent can result in the emergence of collective cell behavior, including cell-sorting, chemoprotection and collective migration. We derived several gemcitabine resistant subclones from the human pancreatic cancer cell line BxPC3 and determined that the observed chemoresistance was driven of a focal amplification of the chr11p15.4 genomic region, resulting in over-expression of the ribonucleotide reductase (RNR) subunit RRM1. Interestingly, these subclones display a rich cell-sorting behavior when cultured as mixed tumor spheroids. Furthermore, we show that chemoresistant cells are able to exert a chemoprotective effect on non-resistant cells in spheroid co-culture, whereas no protective effect is seen in conventional 2D culture. We also demonstrate that the co-culture of resistant and non-resistant cells leads to collective migration where resistant cells enable migration of otherwise non-migratory cells.

## Introduction

It has long been recognized that tumors display rich cellular heterogeneity and that this likely contributes to disease progression, and lack of response to treatment [1] Although most cancers are thought to be clonal in origin, fully developed tumors are typically polyclonal [2,3], displaying significant genetic, epigenetic, and phenotypic heterogeneity. This intra-tumor diversity increases the difficulty of treating cancer, as it makes tumors more robust to perturbations and therapeutic interventions [4–6], and likely contributes to metastasis [7]. Competition and cooperation between cancer subclones likely plays a role in cancer progression, metastasis and chemoresistance [4,8]. In some contexts, aggressive cancer traits may emerge from the interaction between cell populations, i.e. as collective traits, rather than as traits of individual subpopulations [9]. Situations in which traits expressed by a small subpopulation lead to population-wide benefit have been described across ecological systems, such as in the context of bacterial resistance to antibiotics [10]. Similar mechanisms may be relevant to therapy resistance in some tumor cell populations [9]. Within the tumor microenvironment, cytotoxic drugs act as a microevolutionary pressure that selects for drug resistance, often resulting in emergence of chemoresistant clones and disease progression despite intensive treatment. In addition, many cancer therapies are mutagenic [11,12], facilitating the emergence of chemoresistance while also increasing the genetic heterogeneity of tumors. Chemoresistance has been shown to drastically change the phenotype and behavior of cancer cells. Cells with acquired chemoresistance often display other traits of increased aggressiveness, such as increased migration, invasion, and stemness [13–17]. These traits have been shown to be especially relevant in the context of pancreatic cancer resistance to gemcitabine (Gemzar, dFdC) [13–15], the current standard of care.

However, the effect of gemcitabine resistance on collective behavior in heterogeneous pancreatic cancer cell populations has not been studied in detail. The heterotypic interaction between cancer cells from different subpopulations is thought to directly contribute to migration and invasion [18]. Leader-follower dynamics, where a more invasive cell-population paves the way for less invasive cells, have been observed in tumor cell populations [19,20]. Collective invasion of heterogeneous cancer cell populations has been shown to give rise to polyclonal metastasis in breast cancer models [21], which is also supported by data from models of pancreatic cancer [22], underscoring the importance of studying processes that drive the development of collective behavior in cancer tumors.

Gemcitabine is a nucleoside analog, and induces cell death by terminating DNA replication and inhibiting the ribonucleotide reductase (RNR) enzyme complex. The gemcitabine prodrug requires intracellular phosphorylation, and the cytotoxic activity is exerted by its di- and triphosphorylated metabolites (dFdCDP and dFdCTP). Overexpression [23,24] and copy-number amplification [25] of the large subunit of RNR (RRM1) has been associated with the development of chemoresistance *in vitro* and in patients [26]. As the reaction of gemcitabine diphosphate with an active RNR complex results in the irreversible binding of the gemcitabine ribose to RRM1, gemcitabine functions as a suicide inhibitor of RNR [27]. Molecular studies have indicated that overexpression of RRM1 may lead to detoxification of gemcitabine as RNR activity can be regained by exchanging the inactivated RRM1 subunit for active RRM1 [28].

Because of the high lethality of pancreatic cancer, the ubiquity of gemcitabine treatment, and the potential for polyclonality and collective behavior in gemcitabine-treated pancreatic cancer cells, we set out to create an *in vitro* 3D tissue engineering model of microtumor formation, chemoresistance and migration/invasion, building on work by others [20,29,30]. We set out to determine whether the interaction between closely related gemcitabine resistant and non-resistant pancreatic cancer cell populations can give rise to collective resistance to chemotherapy and collective migration. In this in vitro model, we show how the acquisition of chemoresistance in one cancer subclone can increase the aggressiveness of the cancer cell population as a whole. Specifically, we show that acquired chemoresistance drives spatial heterogeneity in pancreatic spheroid microtumor co-cultures, and that chemoresistant cells can exert population-wide chemoprotection and facilitate collective cell migration.

## Materials and methods

### Cell culture and generation of gemcitabine resistant cell lines

BxPC3 and PANC-1 cells were obtained from ATCC. Cell lines were cultured in a humidified incubator with 5% CO_2_ at 37°C as recommended by ATCC (BxPC3: 10% FBS RPMI and PANC-1: 10% FBS DMEM) supplemented with 100 units penicillin, 0.10 mg streptomycin and 0.25 g amphotericin B per ml. Gemcitabine resistant BxPC3 subclones were selected using chronic, low-dose drug treatment, starting at 8 nM (just below the IC50-value for BxPC3 cells). Gemcitabine treated cells were passaged into plates with a range of higher gemcitabine concentrations when confluent and resistant clones emerging after near-complete cell death were isolated and expanded. After establishing, the gemcitabine resistant clones were routinely passaged without gemcitabine, with no observable loss of resistance for over 20 passages. Resistant clones were isolated by colony picking.

### MTT viability assay

The 2D IC50 values for gemcitabine were determined using MTT assays. Cells were seeded at 5,000 cells per 96-well in 200 μl complete media. After adhering overnight, the media was replaced with 150 μl/well of an 11-point dilution curve of each compound with 6 technical replicate wells per condition, including untreated cells and media-only controls. After 96 hours of culture, 16 μl of 5 mg/ml sterile filtered MTT (3-(4,5-dimethylthiazol-2-yl)-2,5-diphenyltetrazolium bromide), Affymetrix) was added per well. Plates were incubated for 4 hours and then centrifuged at 1000xg for 5 min to pack formazan crystals on the well bottom. Media was replaced with 200 μl of acidified isopropanol (50mM HCl and 0.1% Triton X-100 in isopropanol). After complete dissolution of formazan crystals, plates were read at 560nm and 690nm using a plate reader (Biotek). The relative viability for each drug concentration was calculated by subtracting absorbance at 690nm from 560nm, normalizing to the untreated wells, and averaging the 6 technical replicates. Reported n refers to experimental replicates, each representing 6 technical replicates per concentration and cell type. Aggregated MTT viability data can be seen in S1 Fig. Non-linear, three parameter dose-response curves were fitted to each experimental replicate using GraphPad Prism (v9.0.0) and IC50 values were extracted from the fitted curves. Statistical significance was determined using GraphPad Prism.

### CytoTox Red cytotoxicity assays

To assess cell death in response to gemcitabine in 2D cell culture we used automated image analysis on the IncuCyte S3 platform. Cells were seeded and treated in the same manner as for the MTT assay. After 96 hours of gemcitabine incubation, CytoTox Red was added to a final dilution of 1:12,000, without disturbing dead cells, and incubated with the cells at 37°C for 30 min. Relative cell death was estimated by determining the CytoTox Red positive area per well and normalizing to the corresponding MTT viability data.

### Determination of subclone doubling time

To estimate the subclone doubling time, we used automated image analysis on the IncuCyte S3 platform. BxPC3, BxGR-80C and BxGR-360C cells were seeded at 5000 cells per 96 well and imaged every 4 h for a total of 180 h. 12 technical replicates were analyzed per subclone and time point, and a total of 3 experimental replicates were performed. The IncuCyte confluency algorithm was trained to determine confluency as a function of time and the doubling time was determined by fitting the logistic growth function to the confluency data for each experimental replicated in GraphPad Prism (v9.0.0). Statistical significance was determined using GraphPad Prism.

### RNA isolation and mRNA-sequencing

BxPC3, BxGR-80C, BxGR-120C and BxGR-360C cells were seeded at 400,000 cells per 60 mm dish and were allowed to grow for 48 hours. Total RNA was isolated using the RNeasy kit according to manufacturer’s instructions (Qiagen). The sample RQN values were measured on a Bioanalyzer (Agilent), and determined to be 9.8 or higher for all samples. 1 μg total RNA was used to generate stranded, poly-A selected NGS libraries using the NEBNext Ultra Directional RNA kit. Indexed libraries were sequenced on a NextSeq 500 sequencer (75 cycles, single-end), generating a minimum of 24 million reads mapping to annotated genes per library. Reads were aligned and quantified using the Tuxedo Suite pipeline, and cuffdiff2 was used to identify differentially expressed genes. Heatmap and hierarchical clustering of samples based on differentially expressed genes was performed in R using the heatmap.2 function on log2(FPKM+1) values. Raw sequencing data has been deposited at ArrayExpress (accession: E-MTAB-11186). Library preparation, sequencing and basic bioinformatic analysis was performed by the Cornell Transcriptional Regulation and Expression Facility.

### Western blotting

Western blotting was performed using a standard protocol. Briefly, cells were seeded at 500,000 cells/60 mm dish 48 hours prior to lysis. Cells were lysed in 100 μl lysis buffer (50 mM Tris, 1% (v/v) Triton X-100, 150 mM NaCl, 2mM PMSF, 1mM sodium orthovanadate and protease inhibitor cocktail, Santa Cruz Biotechnology). Lysates were quantified with the DC protein assay (Bio-Rad), heat-treated at 95°C in Laemmli sample buffer (2% SDS, 2% 2-mercaptoethanol, 0.1% bromophenol blue, 10% glycerol, 62.5 mM Tris-HCl) for 5 minutes. For routine western blotting, 10–15 μg total protein was then loaded per well in 4–20% Tris-Glycine gradient polyacrylamide gels (Thermo). Following SDS-PAGE, proteins were transferred to 0.45 μm PVDF blotting membranes (Millipore). For staining, the membranes were blocked for 2 hours in 3% BSA (w/v) in TBST. Blots were stained with primary antibodies over night at 4°C with constant rocking. Primary antibodies used: anti-RRM1 (EPR8483, abcam), anti-RRM2 (N1C1, GeneTex), anti-RRM2B (AF3788, R&D Systems), anti-dCK (Bethyl), anti-E-cadherin (24E10, CST), anti-Vimentin (D21H3, CST), anti-N-Cadherin (D4R1H, CST), anti-Snail (C15D3, CST), anti-ZEB1 (D80D3, CST), anti-STIM1 (Bethyl), anti-RhoG (1F3 B3 E5, Biolegend), anti-β-actin (D6A8, abcam). All primary antibodies were used at 1:2000 dilution. Following washing, the membranes were stained with HRP-conjugated secondary antibodies (1:2000 dilution) for 1 hour at room temperature. Blots were developed with Western Lightning Plus Enhanced Chemiluminescence Substrate (Perkin Elmer) and imaged on a Chemigenius bio-imager or using X-ray film. To estimate relative RRM1 abundance we performed western blotting on serial dilutions of total protein lysates from BxPC3, BxGR-80C and BxGR-360C to avoid saturation of the membrane. Relative RRM1 abundance in BxGR-80C and BxGR-360C was estimated by determining the total chemiluminescent RRM1 band intensity, normalizing to total amount of protein loaded (5 μg for BxPC3, 2 μg for BxGR-80C and 0.7 μg for BxGR-360C) and to BxPC3 control samples. Densitometry was performed in ImageJ. For visualization of the inactivated RRM1 (RRM1*) band, cells were incubated with indicated concentrations of gemcitabine for 24 hours prior to western blotting analysis.

### Copy number analysis

For each replicate, DNA was isolated from pellets containing one million cells using a QIAamp DNA micro kit (Qiagen), following the supplier’s protocol, with the addition of an RNase A treatment step. Isolated DNA was stored at -80°C prior to copy-number analysis. Immortalized HUVEC E4 cells were included as a diploid reference control. A predesigned TaqMan RRM1 qPCR CNV assay (Hs02671698cn, Thermo) was used with a TaqMan RNseP qPCR CNV internal reference assay (Thermo). Copy-number analysis was performed using a ViiA 7 qPCR machine, following the supplier’s instructions. Each sample was analyzed with three technical replicates per analysis and each analysis was repeated three times. To determine the RRM1 copy-number for each cell line and sub-clone, the Ct values were extracted from the amplification plots, a ΔCt value was determined for each sample (ΔCt = Ct_RRM1_ –Ct_RNaseP_), and by assuming exponential amplification a relative copy-number was calculated (n_rel_ = 2^-ΔCt^). An absolute copy-number was calculated by normalizing to the HUVEC diploid control (n_abs, subclone X_ = n_rel, subclone X_/n_rel, HUVEC_ x 2). The amplification plots were consistent with a diploid copy-number of 2 for RNseP in all samples, indicating that it is an appropriate control for the assay. Statistical significance was determined in GraphPad Prism.

### shRNA and siRNA knock-down experiments

For shRNA-mediated knock-down experiments we cloned RRM1 targeting and non-targeting shRNA sequences into the pLKO.3G (addgene, #14748) backbone using EcoRI/PacI digestion, oligo annealing and T4 ligation. We used the following shRNA sequences: RRM1-shRNA_A 5’-CCCACAACTTTCTAGCTGTTT-3’ (TRCN0000038964), RRM1-shRNA_B 5’-CCAATCCAGTTCACTCTAAAT-3’ (TRCN0000038968), non-targeted control 5’-CCTAAGGTTAAGTCGCCCTCG-3’. Lentiviral particles were generated using psPAX2 packaging vector (addgene #12260) and pCMV-VSVG envelope vector (addgene # 8454). BxPC3, BxGR-80C and BxGR-360C cells were transduced in the presence of 8 μg/ml polybrene, wells containing less than 10% GFP expressing cells were sorted for GFP. GFP sorted cells were expanded and used in knock-down experiments.

For siRNA-mediated knock-down, cells were seeded at 400,000 cells/6-well and allowed to adhere over-night. Media was then replaced with 2 ml complete media. Cells were then transfected with 125 pmol siRNA per million cells using Lipofectamine RNAiMAX (Thermo) following the manufacturer’s instruction. For western blotting the cells were allowed to incubate with the transfection agent for 72 hours, followed by lysis and western blotting with the standard procedure described above. Degree of RRM1 knock-down was estimated using densitometry in ImageJ. For MTT assays, cells were seeded at 5,000 cells per well after 24 hours of incubation with the transfection agent following the standard procedure described above. Gemcitabine IC50 values were determined as described in the MMT cytotoxicity assay section. For each experiment, 6 technical replicates were quantified per gemcitabine concentration and cell type, and 3 experimental replicates were performed. Pre-designed RRM1-specific or non-targeting Stealth siRNAs (Thermo) were used for knock-down: RRM1 (HSS109388, 5’-AAGAUCUGCUUAUUCAGUAACUGGG-3’).

### Quantitative reverse transcription PCR (qPCR)

RNA was isolated from cultured cells using an RNeasy Mini Kit (Qiagen) and reverse transcribed using the SuperScript™ III First-Strand Synthesis System (Thermo Fisher). qPCR was performed using Power SYBR Green Master Mix on a Quantstudio 3 real-time PCR system. Relative RRM1 mRNA abundance was determined using the ΔΔC_t_ method with GAPDH as housekeeping gene. The following primers were used: RRM1 F: 5’-ACCAGCAAAGATGAGGTTGC-3’ and R: 5’-GGGGCGATGGCGTTTATTTG-3’, GAPDH F: 5’-AGCCACATCGCTCAGACAC-3’ and R: 5’-GCCCAATACGACCAAATCC-3’.

### Immunofluorescence

Cells were seeded in tissue-culture treated optical-bottom 96-well plates (Thermo) and allowed to grow for 24 hours. Following washing with PBS, cells were fixed with 2% formaldehyde in a PBS/PHEM buffer (60 mM PIPES, 25 mM HEPES, 10 mM EGTA, 2 mM MgCl2) for 15 minutes. Cells were then blocked with 10% normal goat serum for 45 minutes, followed by permeabilization with 0.1% saponin for 5 minutes, when necessary. Following permeabilization, cells were incubated with primary antibodies overnight at 4°C: anti-E-cadherin (24E10, CST, 1:400), RRM1 (EPR8483, abcam, 1:400) and cytokeratin (C11, Biolegend, 1:400). Cells were then incubated with Alexa Fluor 488, 568 or 647-conjugated secondary antibodies (1:1000, Life Technologies) for 1 hour at room temperature. For phalloidin staining, cells were incubated with phalloidin conjugated to CruzFluor488 (1:1000, Santa Cruz Biotechnology) in 1% BSA/PBS. Cells were counterstained with 1 μg/ml DAPI (Sigma-Aldrich) for 15 minutes. Representative images were acquired using a Nikon Eclipse TE2000-U fluorescent microscope or using a Zeiss i880 confocal/multiphoton microscope. Cell-cell junction enrichment of E-cadherin was calculated using automated image analysis in CellProfiler, see example analysis in S2 Fig. Cell nuclei were identified as primary objects using their DAPI signature. Cell outlines were identified by propagation, using E-cadherin staining and with nuclei as seeds. Expanded and shrunken cell outlines were defined by expanding and shrinking the cell outline by 2 and 7 pixels, respectively. The cell edge was defined as the area resulting from the subtraction of the shrunken from the expanded outline. The integrated mean E-cadherin intensity was determined and averaged for the edge regions and shrunken cytoplasm for each cell. Three independent fields of view were analyzed per cell type, each representing a minimum of 160 cells.

### Attachment-free spheroid cultures

Spheroids were generated by allowing cells to self-assemble in attachment-free culture. Agarose was used to create cell-attachment-free wells in conventional 96-well plates. 1.5% (w/v) agarose was dissolved in deionized water by heating and sterilized by autoclavation. 50 μl melted agarose was added to 96-well plates, spontaneously forming a concave shape collecting cells in the center of the well. When solidified, 100 μl cell-suspension was then added per well and plates were incubated at 37°C. Using this approach, a single spheroid self-assembles in each well. A total of 2,000 cells were seeded per well. When appropriate, a final concentration of 5 μM of Rac1 inhibitor (EHop-016, Cayman), 10 μM FAK inhibitor III or 20 μM ROCK inhibitor (Y-27632, Cayman) was added after seeding cells.

### Spheroid cytotoxicity and viability assays

Spheroids consisting of BxPC3 and BxGR-360C mono-, and co-cultures were seeded in attachment-free culture at a total number of 2000 cells per spheroid. 48 hours after seeding spheroids, gemcitabine was added at indicated final concentrations. Spheroids were incubated with gemcitabine for 6 days. To assess spheroid cell-death through imaging, propidium iodide (4 μg/ml) and/or Calcein AM (4 mM) was incubated with the spheroids for 30 minutes at 37°C without disturbing dead cells. The wells were then imaged using a Nikon Eclipse TE2000-U fluorescent microscope or on the IncuCyte S3 platform. For quantitative analysis, the spheroid-containing area was determined for each well using automated analysis on the bright field images on the IncuCyte S3 platform. Spheroid cell death was estimated by calculating the integrated PI intensity across the spheroid area for each well and normalizing to untreated controls, see S3 Fig for example analysis. Four technical replicates per concentration were quantified, and four experimental replicates were performed. To assess loss of spheroid viability in response to gemcitabine, we used the CellTiter-Glo 3D (Promega) assay following the manufacturer’s instructions. Collected chemiluminescent intensities were normalized to untreated controls. 2–4 technical replicates per concentration were quantified, and three experimental replicates were performed. When appropriate, cells were treated with RRM1 or non-targeting siRNA, as described above, for 24 hours prior to spheroid seeding.

### Quantification of chemoprotection

To quantitatively determine absence or presence of chemoprotection in 2D and spheroid culture, we compared the observed viability to the expected value, calculated from the corresponding mono-cultures. To determine the expected 2D viability of BxPC3 and BxGR-360C co-cultures as a function of gemcitabine concentration, we calculated the simple linear combination of the normalized MTT viability values for pure BxPC3 and BxGR-360C culture, taking the culture ratio into account, i.e. Expected Viability_BxPC3 3:1 BxGR-360C, X μM_ = (3 x Viability_BxPC3, X μM_ + Viability_BxGR-360C, X μM_)/4. Expected spheroid viabilities were calculated analogously using the values from the CellTiter-Glo 3D assay. The chemoprotection coefficient (Observed/Expected viability) was then calculated as C_O/E_ = Observed Viability_BxPC3 3:1 BxGR-360C, X μM_/Expected Viability_BxPC3 3:1 BxGR-360C, X μM_.

### Multiphoton imaging of spheroid cell sorting

Cells were seeded in complete media at 100,000 cells/24-well and were allowed to adhere over-night. Cells were then labelled with either CellTracker Green or CellTracker Red (Thermo) at 10 μM for 30 min. After washing with complete media, spheroids were seeded normally. Spheroids were imaged on using a Zeiss i880 confocal/multiphoton microscope. 2-photon excitation was done with 800 nm light and the fluorophores were separated by emission.

### Determination of cell-sorting in spheroids

To quantitatively determine cell-sorting in BxPC3 and BxGR-360C co-culture spheroids we performed automated image acquisition on the IncuCyte S3 platform. Spheroids consisting of a majority of unlabeled BxPC3 cells, mixed with 25% CellTracker Red labelled BxPC3 or BxGR-360C cells, as well as labeled spheroids mono-cultures were generated as described above. The radial distribution profile of CellTracker Red intensity was determined for the extracted images using the Radial Profile plugin in ImageJ, see S5 Fig. The average spheroid radius for each spheroid type was defined as the distance from the center that encloses 95% of the total intensity for each spheroid type, and the center intensities were normalized to 1. The spheroid edge region intensity was defined as the normalized intensity at normalized distance to center = 0.77. 20 spheroids were analyzed per spheroid type. Statistical testing was performed in GraphPad Prism.

### Spheroid cryosectioning and immunofluorescent staining

Spheroids were collected and allowed to settle in microcentrifuge tubes on ice. Following washing with ice cold PBS, spheroids were fixed in 4% formaldehyde in a PBS/PHEM buffer (60 mM PIPES, 25 mM HEPES, 10 mM EGTA, 2 mM MgCl2) for 20 minutes. Spheroids were stained with 1% methylene blue for 1 minute to make spheroids visible when cryosectioning. Methylene blue-stained spheroids were embedded in Tissue-Tek O.C.T Compound and flash frozen in liquid nitrogen. Spheroids were stored for a minimum of 48 hours in -80°C before cutting. 8 μm spheroid sections were cut onto glass slides. Following removal of embedding matrix by dipping in deionized water and PBS, slides were stained following the standard immunofluorescence protocol described above.

### Spheroid collagen migration assays

Cell were labelled with CellTracker Green or Red, and spheroids were seeded as described above. After 48 hours of culture, the spheroids were harvested. When appropriate, spheroids were incubated with 10 μg/ml anti-integrin β1 antibody (TS2/16, Thermo) for 30 minutes on ice. Spheroids were then embedded in culture media-supplemented Type I Bovine Collagen (Corning) at a final concentration of 1.5 mg/ml and was allowed to solidify for 1 hour prior to adding complete media. Spheroids were then imaged every 24 hours using a Zeiss i880 confocal/multiphoton microscope or using a Nikon Eclipse TE2000-U fluorescent microscope as described above. To quantify migration, we measured the distance from the spheroid center to the end of the three longest, continuous, BxPC3-containing (CellTracker Green positive), invasive cell sheets for each spheroid. The distances were then normalized to the starting spheroid diameter for each spheroid type. 4 spheroids from each type were analyzed.

## Results

### Generation of gemcitabine-resistant BxPC3 subclones

**Fig 1:**
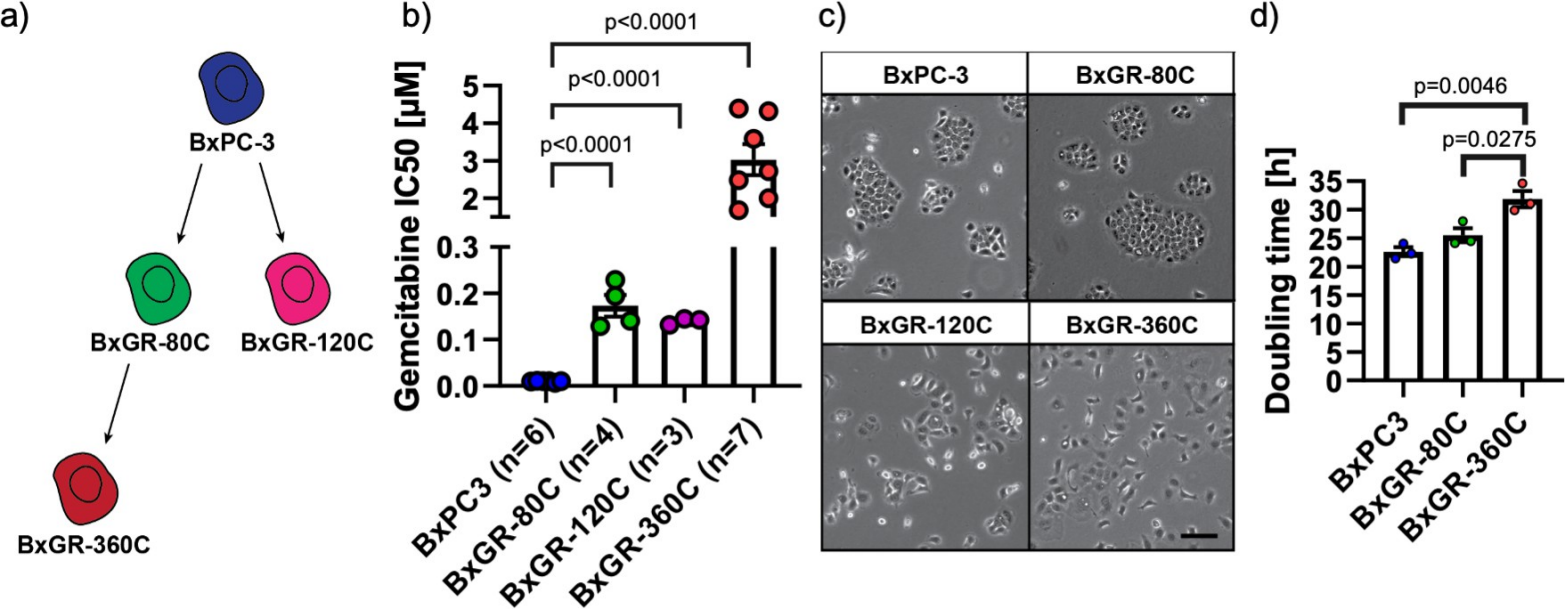
Derivation of gemcitabine-resistant subclones. a) Schematic of gemcitabine-resistant subclones derived from the human pancreatic cancer cell line BxPC3. b) Comparison of gemcitabine IC50-values for BxPC3, BxGR-80C, BxGR-120C and BxGR-360C cells. IC50 values determined from three-parameter non-linear dose-response curves fitted to each independent experiment replicate. n indicates the number of independent experimental replicates. See S1A Fig for aggregated dose-response curves. c) Representative phase contrast images of BxPC3, BxGR-80C, BxGR-120C and BxGR-360C cells 24h after seeding. Scale bar 100 μm. d) Determination of subclonal doubling times in BxPC3 and derived gemcitabine resistant subclones. Doubling-times were determined by fitting a logistic growth function to confluency data acquired using automated image analysis in three independent experimental replicates. See S1B Fig for aggregated growth-curves. b), d) Significance was determined with two-tailed Student’s t-test, error-bars indicate standard error of the mean (SEM).

To investigate the relationship between the acquisition of gemcitabine resistance and associated phenotypic alterations in pancreatic cancer cells, we generated gemcitabine-resistant subclones from the human pancreatic cancer cell line BxPC3 through chronic exposure to increasing concentrations of gemcitabine. First, we determined the BxPC3 gemcitabine IC50-value to be approximately 10 nM using a standard MTT viability assay (S1A Fig). Consequently, gemcitabine treatment was started at 8 nM, just below the IC50-value. At 80 nM gemcitabine we observed near complete cell death, with one resistant clone emerging, which we refer to as BxGR-80C (**Bx**PC3 **G**emcitabine **R**esistant **80** nM **C**hronic exposure). BxGR-80C cells were gradually exposed to higher concentrations of gemcitabine until near-complete death was observed again at 360 nM gemcitabine, with one resistant clone emerging (BxGR-360C). In parallel, we generated the BxGR-120C subclone in the same manner (emerging at 120nM gemcitabine), starting from BxPC3 cells (Fig 1A). The gemcitabine resistance of these subclones was observed to be stable and retained when subcultured without gemcitabine for over 20 passages. Using MTT viability assays, we determined the 96h gemcitabine IC50-values to be approximately 10 nM, 140 nM (14-fold increase), 170 nM (17-fold increase) and 3000 nM (300-fold increase), for BxPC3, BxGR-120C, BxGR-80C and BxGR-360C cells respectively, see Figs 1B and S1A. The BxPC3 and BxGR-80C cells were observed to display similar epithelial morphologies and growth behavior in 2D cell culture (Fig 1C). BxGR-120C and BxGR-360C cells were found to display altered morphology and growth pattern and form loosely associated colonies only after prolonged culture (>48h). These phenotypic alterations were most evident in the BxGR-360C cell cultures, who also grew slower than the other subclones. The doubling-times were determined to be approximately 23, 25.5 and 32 hours for BxPC3, BxGR-80C and BxGR-360C, respectively, see Figs 1D and S1B.

### RNA-seq transcriptional analysis of gemcitabine resistant clones

**Fig 2:**
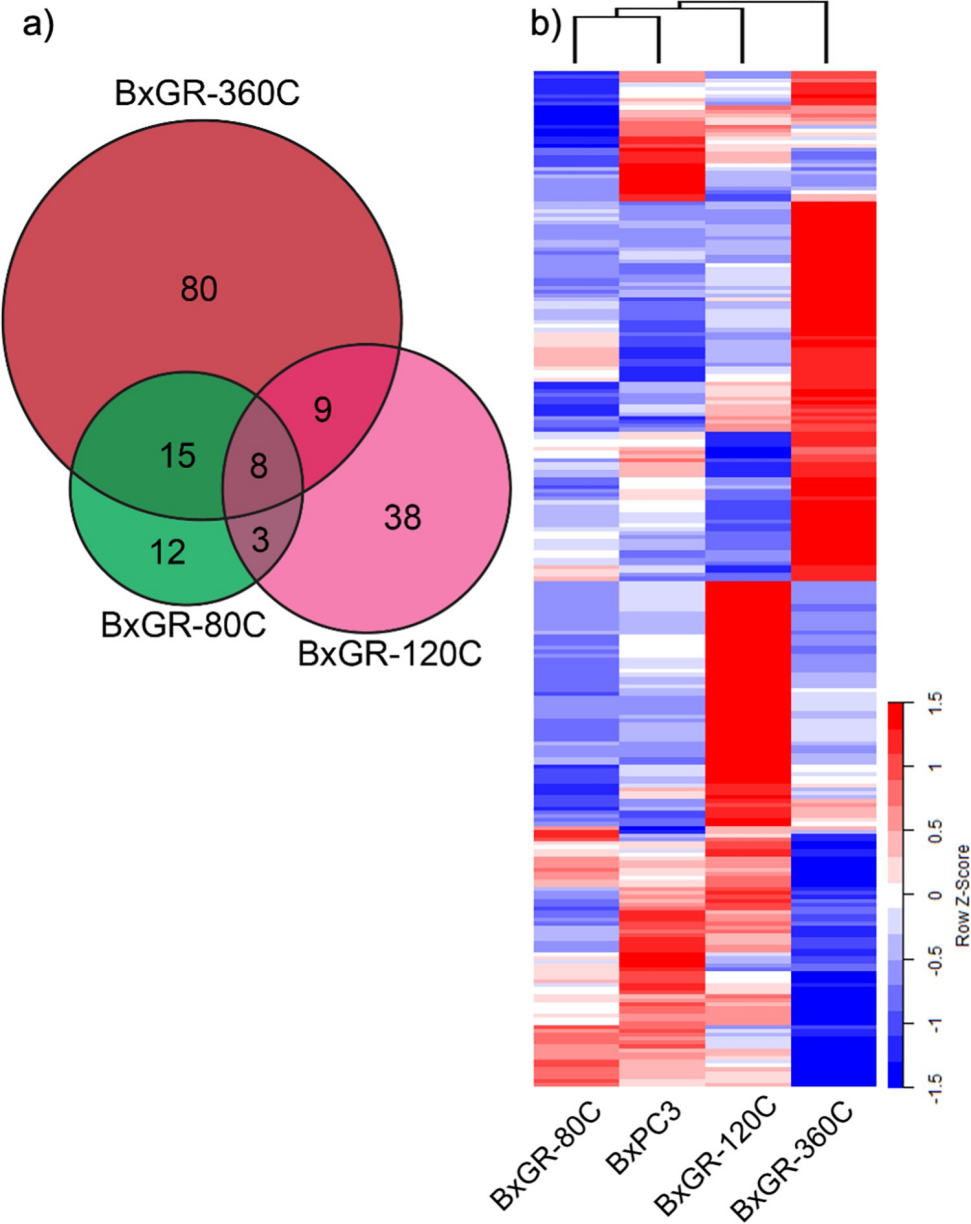
Transcriptional analysis of gemcitabine resistant subclones. a) Venn diagram of differentially expressed genes in resistant clones BxGR-80C, BxGR-120C and BxGR-360C relative to BxPC3 cells. b) Heatmap and unsupervised hierarchal clustering of differentially expressed genes, relative to BxPC3 control.

Previous studies found significant phenotypic alterations, including activation of an Epithelial-Mesenchymal Transition (EMT) program following acquisition of resistance to gemcitabine, suggesting transcriptional reprogramming [13,14]. To investigate this in an unbiased way, we performed mRNA-seq analysis of the BxPC3 cell line and the three derived gemcitabine resistant clones. As can be seen in Fig 2A, 38 genes were differentially expressed in BxGR-80C relative to BxPC3 cells. The corresponding numbers for BxGR-120C and BxGR-360C were 58 and 112 genes, respectively. The largest number of differentially expressed genes (138 genes) was observed between BxGR-120C and BxGR-360C cells. In total, 265 genes were determined to be differentially expressed in one or more of the pairwise comparisons. Unsupervised hierarchical clustering revealed that BxPC3 and BxGR-80C display the most similar expression profiles and that the BxGR-360C expression profile was the most dissimilar (Fig 2B). Next, we performed gene set enrichment analysis (GSEA) looking for enriched gene sets in our gemcitabine resistant subclones relative to the parental BxPC3 cells. Specifically, we evaluated all gene sets contained in the Hallmark, KEGG, REACTOME and GeneOntology Biological Process MSigDB collections. As relatively few differentially expressed genes were found in our analysis, very few gene sets were found to be statistically significantly enriched (p<0.05, FDR<0.15, minimum size = 5). Several interferon gamma and chemokine gene sets were enriched in BxGR-120C cells, whereas BxGR-360C cells were enriched for the GOBP_INNATE_IMMUNE_RESPONSE gene set. The results from this analysis can be found in S1 Table. As gemcitabine resistance has been associated with EMT in previous studies, we were surprised to see than neither subclone was significantly enriched for the HALLMARK_EPITHELIAL_MESENCHYMAL_TRANSITION genes set. However, closer inspection revealed that differential expression of several EMT-associated factors (specifically POSTN, COL3A1, COL5A2, CDH2/N-cadherin, FN1, MXRA5 and BDNF) was found in BxGR-360C relative to BxPC3 cells, but not in either of the other subclones. To look for potential drivers of gemcitabine resistance, we looked for genes that were over-expressed in all resistant subclones, resulting in a list of only 8 genes (S1 Table). Among these, only 2 genes (RRM1 and MAGEB2) were overexpressed. To characterize expressional differences between the two resistance lineages, we looked for genes differentially expressed in BxGR-80C and BxGR-360C relative to BxGR-120C and found that only 3 genes fulfilled this criteria (S1 Table). Among these, RHOG was significantly overexpressed in BxGR-80C and BxGR-360C, but not in BxGR-120C. Our results indicate transcriptomic reprogramming in the gemcitabine resistant subclones, but with relatively few shared features between subclones. Most notably we found universal overexpression of the known gemcitabine resistance-mediator RRM1 in all subclones.

### Gemcitabine resistant subclones display RRM1 overexpression and copy number amplification

**Fig 3:**
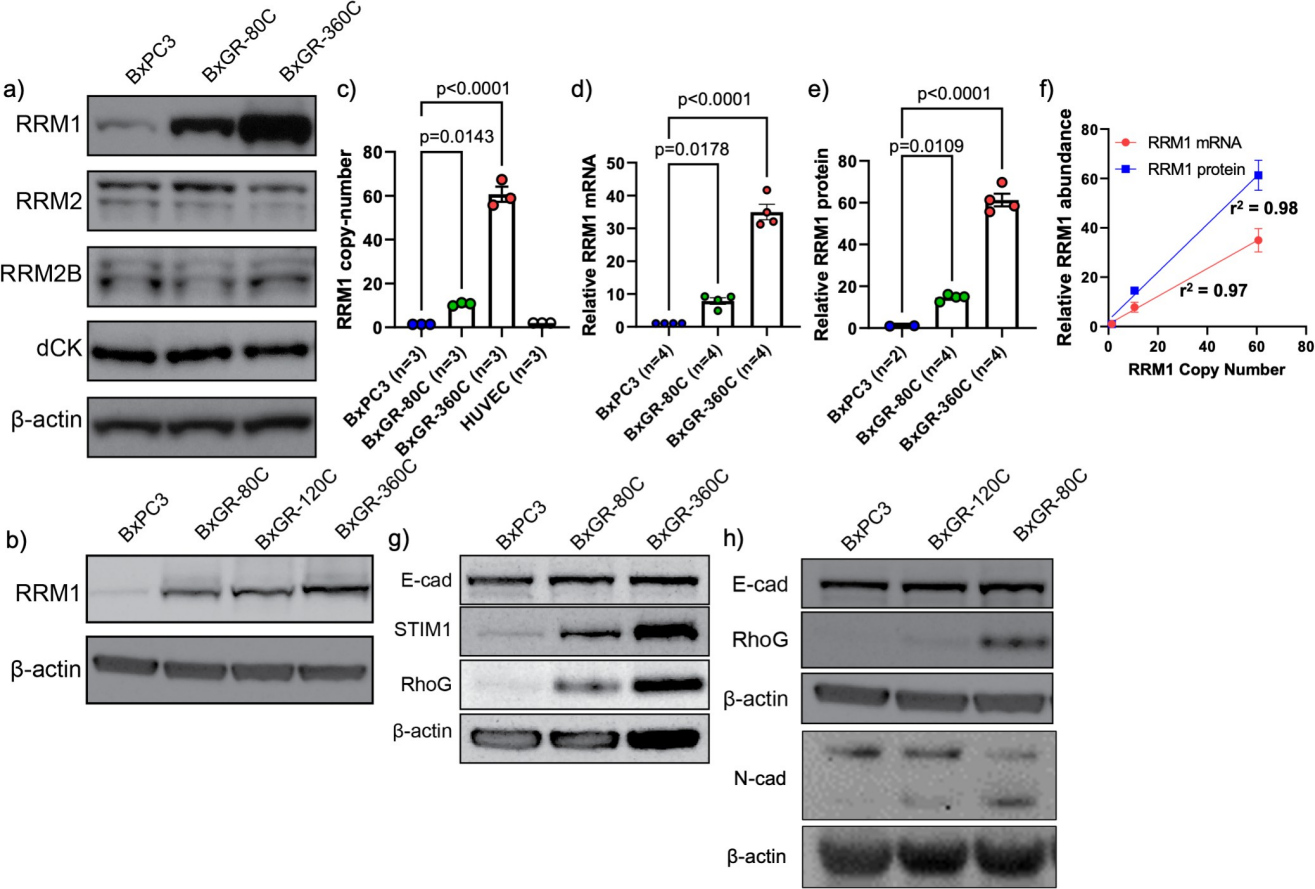
Confirmation of protein-level overexpression and copy number alteration in gemcitabine resistant sub-clones. a-b) Western blotting analysis of RRM1, RRM2, RRM2B and deoxycytidine kinase (dCK) in BxPC3 and derived subclones BxGR-80C, BxGR-120C and BxGR-360C. c) RRM1 TaqMan Copy Number Analysis of BxPC3 and derived BxGR-80C and BxGR-360C subclones. HUVEC cells used as diploid reference control. Three independent DNA samples analyzed per cell type d) Estimation of relative RRM1 mRNA abundance in BxGR-80C and BxGR-360C relative to BxPC3 cells. e) Estimation of RRM1 protein abundance in BxGR-80C and BxGR-360C relative to BxPC3 cells. f) Correlation between RRM1 copy-number, RRM1 mRNA and protein abundance for BxPC3, BxGR-80C and BxGR-360C cells. Solid lines are linear regressions, r^2^ is the coefficient of determination. g-h) Western blotting of E-cadherin, STIM1 and RhoG in BxPC3 and gemcitabine resistant subclones. c-e) Statistical significance performed using one-way ANOVA with Tukey’s multiple comparison test. Error-bars indicate standard error of the mean and n indicates independent samples.

Our transcriptional analysis suggested involvement of RRM1 in the chemoresistance of the BxPC3 subclones. To verify protein-level overexpression, we characterized the expression of RRM1 and other known resistance mediators using western blotting (Fig 3A and 3B). This panel consisted of RNR subunits RRM1, RRM2 and RRM2B, as well as the gemcitabine-activating enzyme deoxycytidine kinase (dCK). Consistent with transcriptional analysis, we found that all the BxPC3 subclones overexpress RRM1, whereas the expression of the other proteins assayed was unchanged.

The RRM1 gene is located in the chr11p15.4 region of the human genome and our transcriptional analysis revealed that BxGR-80C and BxGR-360C, but not BxGR-120C, overexpress five neighboring genes with similar fold overexpression (PGAP2, RHOG, STIM1, TRIM21 and TRIM68, see S1 Table). This pattern suggest chromosomal aberrations and potential copy-number amplification in the chr11p15.4 region in BxGR-80C and BxGR-360C cells. The PGAP2 and RHOG genes are located 420 kbp and 270 kbp upstream of RRM1, respectively. STIM1 is located in a head-to-tail configuration only 1.6 kbp from the 5’ end of RRM1, and TRIM68 located 460 kbp downstream of RRM1. In total the genomic region containing these genes is approximately 925 kbp. Using TaqMan Copy Number Analysis, we determined BxGR-80C and BxGR-360C to have accumulated RRM1 copy-number amplifications of approximately 11- and 61-fold, respectively (Fig 3C). Modal RRM1 copy-number of BxPC3 was determined to be 1.4, indicating baseline chromosome 11 aberrations. We also estimated relative RRM1 mRNA and protein abundance in these cells using qPCR and western blotting. qPCR revealed 8 and 35-fold RRM1 mRNA overexpression in BxGR-80C and BxGR-360C relative to BxPC3, respectively (Fig 3D). Using western blotting, we estimated that the BxGR-80C and BxGR-360C subclones overexpress RRM1 approximately 14- and 60-fold relative to BxPC3 (Fig 3E). Furthermore, we found a high degree of correlation between RRM1 copy-number and mRNA, and between RRM1 copy-number and protein abundance (Fig 3F).

We also confirmed protein-level overexpression of STIM1 and RhoG, displaying the same pattern of overexpression as RRM1 in the BxGR-80C and BxGR-360C cells (Fig 3G) whereas BxGR-120C was found to not overexpress RhoG (Fig 3H). From these observations we conclude that the BxGR-80C and BxGR-360C subclones have acquired significant copy number amplifications of a region of chromosome 11 comprising at least 925 kb, resulting in transcriptional and protein-level overexpression of RRM1.

### RRM1 overexpression drives gemcitabine resistance

**Fig 4:**
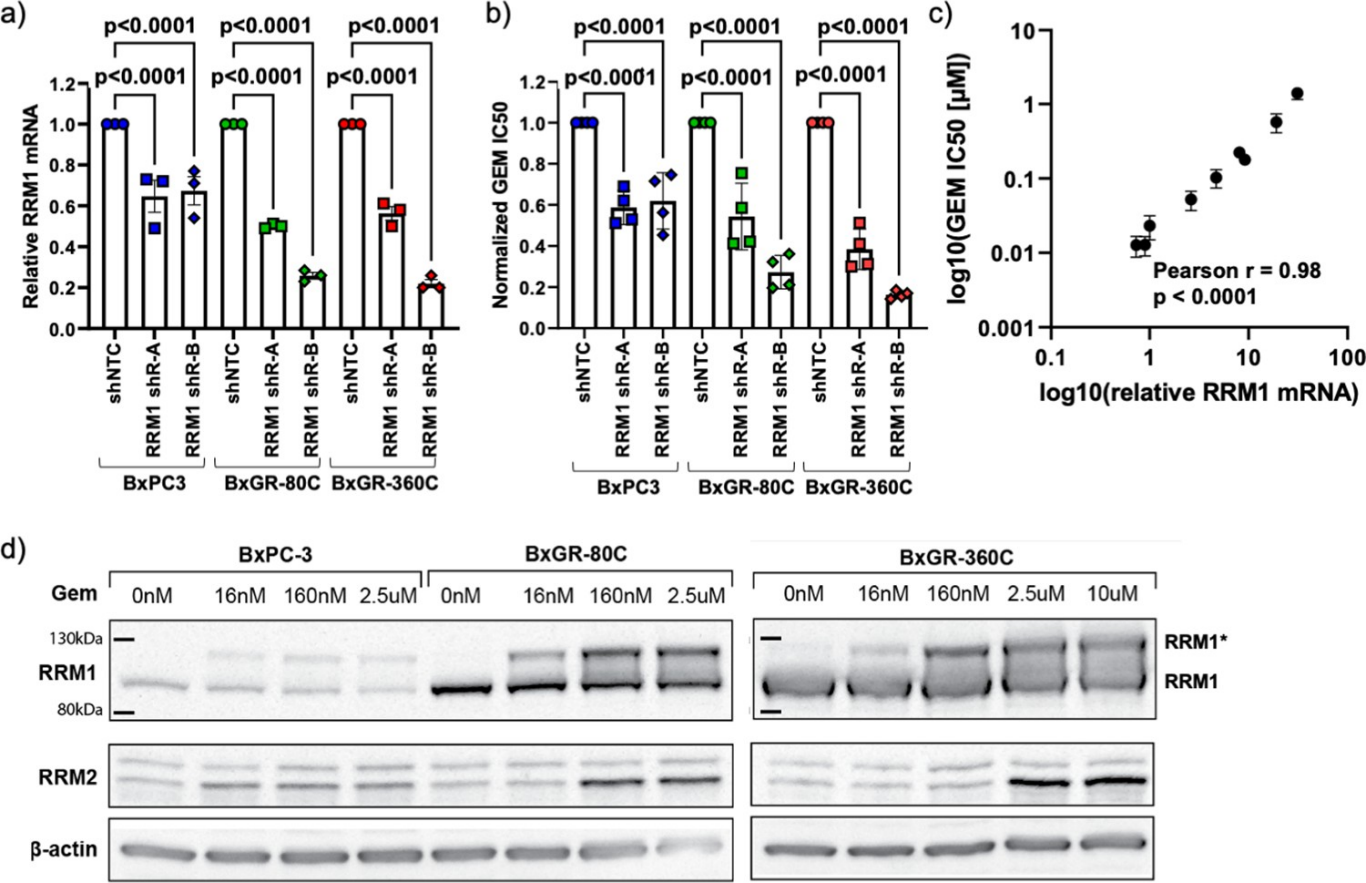
Gemcitabine resistance is driven by RRM1 overexpression. a) Quantification of relative RRM1 mRNA abundance following shRNA-mediated knock-down in BxPC3, BxGR-80C and BxGR-360C cells. Three independent RNA samples analyzed per cell type. b) Relative gemcitabine IC50 values following RRM1 knock-down, normalized to the control for each subclone. c) Correlation between relative RRM1 mRNA abundance and absolute gemcitabine (GEM) IC50 for all RRM1 knock-down cells and controls. d) Western blotting of dose-dependent induction of an RRM1 band with the apparent mass of 110–120 kDa (RRM1*) and RRM2 in gemcitabine treated cells. a-b) Statistical significance tested using one-way ANOVA with Tukey’s multiple comparison test. Error-bars indicate standard error of the mean.

To test if RRM1 overexpression is responsible for the observed gemcitabine resistance, we knocked down RRM1 in BxPC3, BxGR-80C and BxGR-360C cells using lentiviral shRNA constructs and quantified the degree of RRM1 mRNA knock-down and change in gemcitabine IC50 value. shRNA transduction resulted in a variable degree (30–80%) of knock-down relative to the non-targeted controls for each subclone, as measured by qPCR (Fig 4A). We found that RRM1 knock-down resulted in a 33–85% reduction in gemcitabine IC50 values relative to the non-targeted control for each subclone (Fig 4B). We also found a direct relationship between relative RRM1 mRNA abundance and absolute gemcitabine IC50 value across all subclones and knock-downs (Fig 4C), as indicated by a Pearson coefficient of correlation of r = 0.98. These results show that RRM1 is driving resistance in these gemcitabine resistant subclones and that the degree of gemcitabine tolerance is directly proportional to the RRM1 abundance. We also confirmed these results using siRNA-mediated knock-down. BxGR-360C cells with an approximate 50% protein-level RRM1 knock-down relative to siRNA control, as estimated by western blot (S1C and S1D Fig), displayed a partial, yet drastic, resensitization to gemcitabine (S1E and S1F Fig).

It has previously been reported that the inactivated form of RRM1 (RRM1*) [28,31] has an apparent molecular mass of 110-120kDa in SDS-PAGE, rather than the expected 90kDa, allowing for the direct observation of RRM1 inactivation with western blotting [31]. To study the gemcitabine dose-dependence of RRM1 inactivation, gemcitabine-treated cells were assayed for RRM1 and RRM2. We observe dose-dependent induction of an RRM1 band with an apparent mass of approximately 120kDa, in addition to the normal 90kDa band in cells treated with gemcitabine (Fig 4D). The RRM1* band appears to increase in intensity until the approximate gemcitabine IC50 concentration for each particular subclone is reached. At gemcitabine concentrations above the IC50 value, the band intensity appears to be constant, potentially indicating a maximum accumulation of RRM1*. We also observe induction of RRM2 expression at gemcitabine concentrations above the IC50-value for each subclone. Since RRM2 expression peaks in S-phase [27], the observed induction could be a result of accumulation of cells in S-phase or possibly upregulation in response to lack of RNR-activity. These results indicate that RRM1-overexpressing cells may be able to sequester and inactivate gemcitabine in the cellular microenvironment and show that RRM1 overexpression likely is the main driver of gemcitabine resistance in these cells.

### Gemcitabine-resistant cells display an invasive phenoptype

The highly gemcitabine-resistant BxGR-360C subclone displays a significantly altered morphology and growth pattern, see Fig 1C. Our transcriptional analysis gave some indication of potential EMT in the BxGR-360C cells, but not in the other subclones. To investigate this finding further, we stained BxPC3 and BxGR-360C cells for canonical EMT markers E-cadherin, N-cadherin and vimentin (S1G Fig). However, while no overt differences were observed for vimentin and N-cadherin staining (despite transcriptional analysis indicating N-cadherin over-expression), we noted a markedly different E-cadherin staining pattern in BxGR-360C cells. Furthermore, western blotting for E-cadherin and N-cadherin revealed no clear differences in total protein levels between BxPC3, BxGR-80C and BxGR-360C (Fig 3G and 3H) and no expression of canonical EMT transcription factors Snail and ZEB1 was detected in the subclones (see Supporting Information for blot images). To investigate potential E-cadherin dysregulation, we used image analysis to determine that, despite expressing the same level of total E-cadherin, BxGR-360C cells display a significantly lower level of E-cadherin enrichment at cell-cell junctions relative to BxPC3 cells, see S1H, S1I and S2 Figs. E-cadherin dysregulation has previously been described as a key characteristic of cancer cell invasiveness [32,33]. BxGR-360C cells were also found to form markedly more and larger lamellipodia and filopodia, as judged by phalloidin/F-actin staining (S1H Fig), consistent with a more invasive phenotype. In summary, our results indicate that some gemcitabine resistant cells, in particular the BxGR-360C subclone, display an invasive phenotype, but with no clear indication of canonical EMT.

### Cancer cells display increased gemcitabine tolerance in 3D spheroid cultures

**Fig 5:**
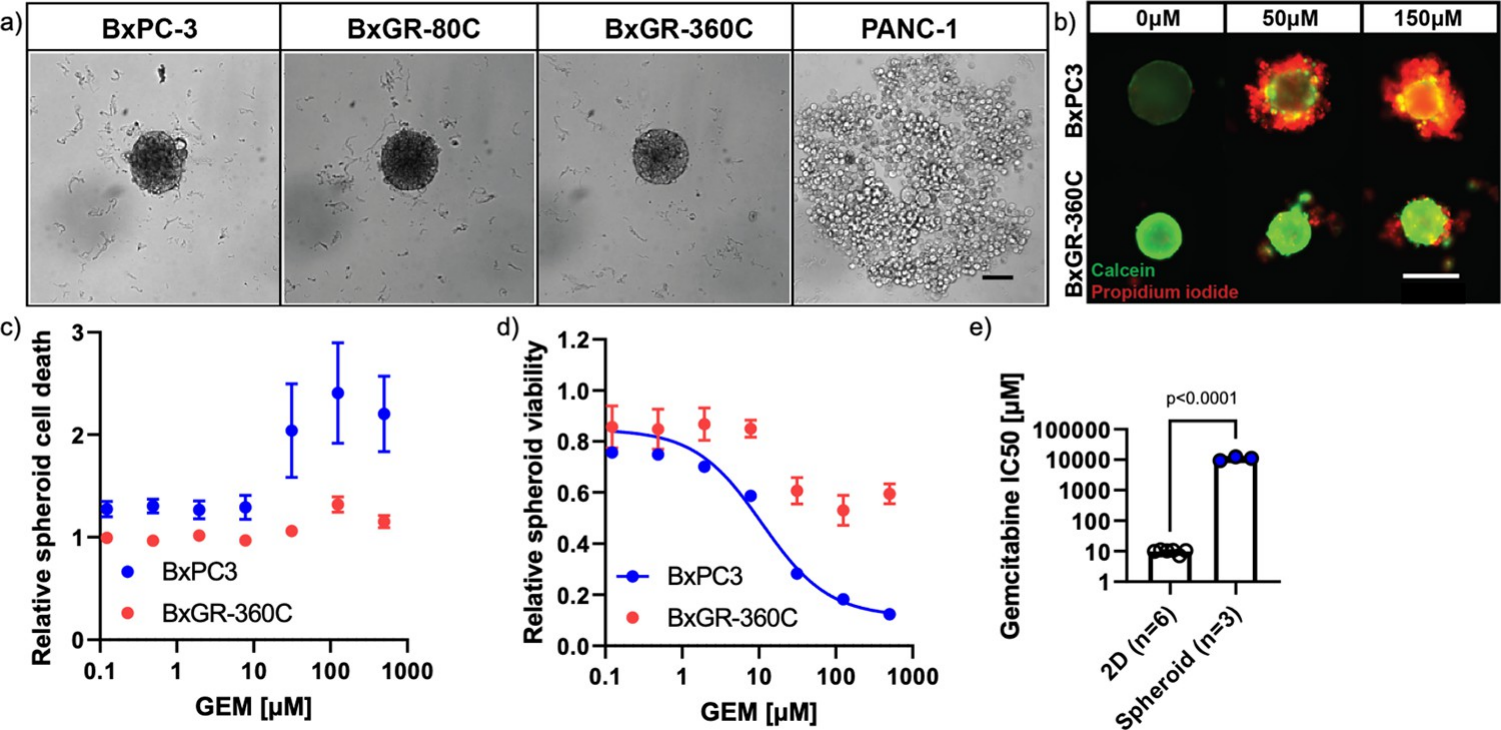
Spheroid formation. a) Spontaneous spheroid formation in BxPC3-derived subclones compared to PANC-1 cells. A total of 2000 cells seeded per well. Scale bar 100 μm. b) Live/dead labeling of spheroid cultures incubated with various concentrations of gemcitabine for 6 days, using Calcein AM (live cells, green) and propidium iodide (dead cells, red). Scale bar 200 μm. c) Quantitation of dead cells in response to gemcitabine incubation (6 days) as measured by PI uptake and automated image analysis. Four independent experimental replicates. Error bars indicate SEM, see S3 Fig for analysis pipeline. d) Determination of spheroid viability in response to gemcitabine incubation (6 days) using CellTiter-Glo 3D across three independent experimental replicates. Error bars indicate SEM. Three-parameter non-linear dose-response curve determined for BxPC3 spheroids. No curve was fitted to BxGR-360C spheroids as only limited loss of viability was observed. e) Estimation of gemcitabine IC50-values of BxPC-3 cells grown in 2D and in spheroids. Gemcitabine IC50-values determined from three-parameter non-linear dose-response curves fitted to independent MTT (2D) or CellTiter-Glo 3D (spheroid) assay experimental replicates. Error bars indicate SEM. Statistical significance performed using two-tailed Student’s t-test. n indicates independent experimental replicates.

Phenotypic alterations associated with gemcitabine resistance may significantly alter the behavior of cancer cells in the tumor microenvironment. To enable study of collective cellular behaviors and the interaction of cancer cell populations in a three-dimensional, tumor-mimicking microenvironment, we developed an attachment-free culture platform for spheroid microtumor generation, based on previously described methods [20]. Using this platform, we found that the gemcitabine-resistant BxPC3 subclones retain the ability to form spheroids within 24 hours of seeding, whereas PANC-1 cells do not (Fig 5A).

To estimate the gemcitabine tolerance of cells grown as spheroids, we developed a live/dead assay based on Calcein AM and propidium iodide (PI) labeling. Gemcitabine treatment of BxPC3 spheroids at 50 and 150 μM for 6 days resulted in the accumulation of dead (PI-labeled) cells on the spheroid surface (Fig 5B).

To allow for quantitative analysis, we implemented automated spheroid image acquisition and analysis using the IncuCyte S3 platform. To measure the induction of cell death in response to gemcitabine dose, we extracted the integrated PI intensity per spheroid from these image sets. As can be seen in Figs 5C and S3, the results revealed that at gemcitabine concentrations greater than approximately 10 μM, BxPC3 spheroids displayed markedly increased cell death, whereas BxGR-360C spheroids were largely unaffected. To measure loss of spheroid viability as a function of gemcitabine dose, we used the CellTiter-3D chemiluminescent assay. Using fluorescent imaging and chemiluminescent detection allowed us to assay cell death and spheroid viability on the same spheroid wells. BxPC3 spheroids displayed a sigmoidal gemcitabine viability response, and the IC50 value was determined to be approximately 11 μM, (Fig 5D). Thus to our surprise, in our culture system, BxPC3 cells display a 1100-fold higher gemcitabine tolerance in spheroid culture compared to conventional 2D culture (Fig 5E). BxGR-360C displayed somewhat reduced viability at higher gemcitabine concentrations, but the viability never decreased below 50% of untreated control, even at 500 μM gemcitabine (Fig 5D). This effect may be due to the decreased cellular growth-rate often observed in 3D culture [34] and limited drug transport into spheroids [35,36].

### Resistant cells preferentially localize to the outer layer of spheroid co-cultures

In some cases, cellular co-cultures spontaneously arrange into segregated domains through a process referred to as”cell-sorting’’. Cell-sorting has been observed in physiological processes such as embryogenesis [37–40], as well as *in vitro* [36,40–42]. The resulting distribution of cells is thought to be governed by the interplay of adhesive and contractile cellular forces [39–41,43]. The spontaneous organization of cell populations into core-shell type aggregates, where one cell type dominates in the core and another dominates the shell, have been observed in a variety of contexts [36,41,44–46].

**Fig 6:**
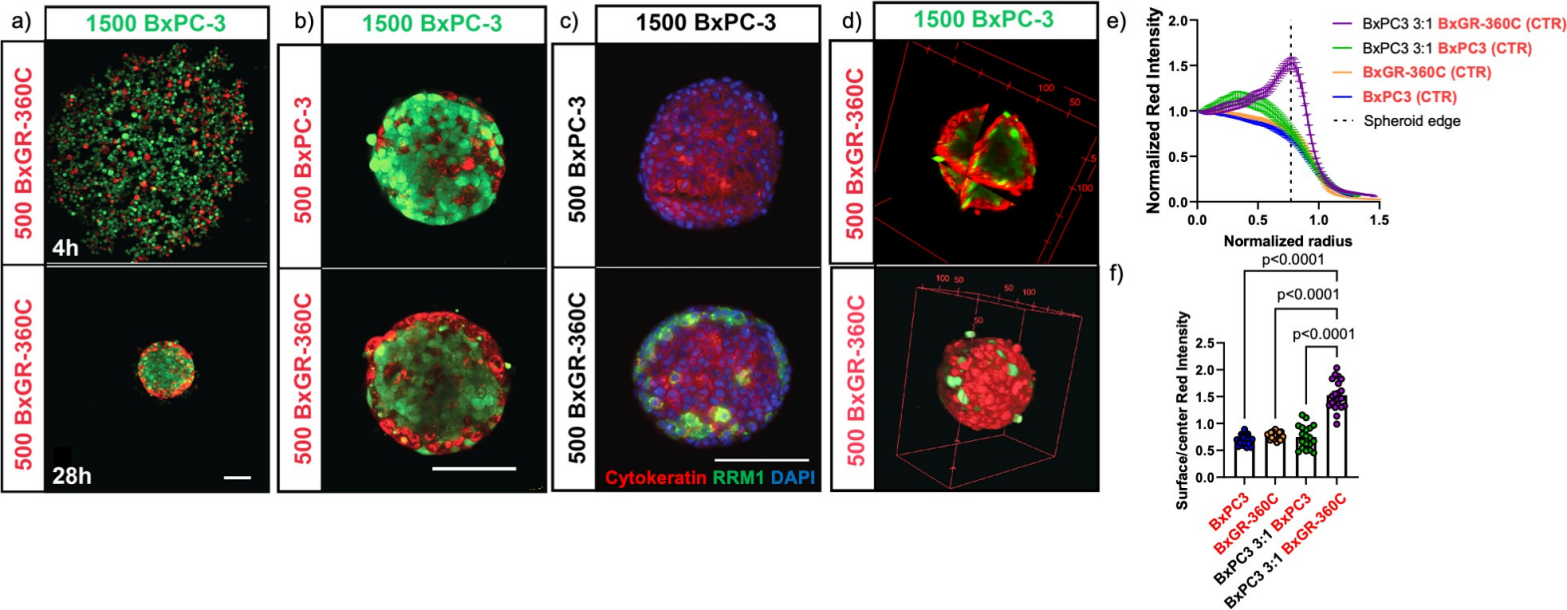
Cell sorting in co-culture spheroids. a) (top) Multiphoton microscopy reveals that a homogeneously mixed and loosely associated network of gemcitabine resistant (BxGR-360C, red, 500 cells) and sensitive cells (BxPC3, green, 1500 cells) forms within 4 hours of attachment-free culture, (bottom) after 28 hours the cellular network has contracted into a spheroid with the resistant cells localized to the surface. Scale bar 100 μm. b) Preferential localization of resistant cells to the outer shell of co-culture spheroids after 48 hours. Scale bar 100 μm. c) Cryosectioning followed by immunofluorescent staining of RRM1 (green) and cytokeratin (red) showing surface localization of RRM1-overexpressing cells, 8 μm thick section, DAPI (blue). Scale bar 100 μm. d) Three-dimensional rendering of a mixed spheroid with near complete surface-coverage of resistant BxGR-360C cells. (top) Cross-sections along major axes, (bottom) three dimensional surface rendering e) Radial distribution of normalized CellTracker Red (CTR) intensity across various single-clone and heterogeneous spheroids revealing surface enrichment of BxGR-360C cells when co-cultured with BxPC3 cells at a 3:1 ratio (BxPC3 3:1 BxGR-360C (CTR)). Figure legend indicates which cells were unlabeled or labeled with CTR prior to seeding. x-axis shows distance to center normalized to spheroid radius. A total of 2000 cells seeded per spheroid. 20 spheroids analyzed per spheroid type. Error bars indicate SEM. f) Ratio of edge to center CTR intensity across various single-clone and heterogeneous spheroids. A total of 2000 cells seeded per spheroid. 20 spheroids analyzed per spheroid type. Spheroid edge defined as the distance when the normalized distance to center equals to 0.77. Error bars indicate SEM. Statistical analysis performed with one-way ANOVA with Tukey’s multiple comparison test. a-d) Shows representative images from a minimum of 3 experimental replicates.

To determine if the phenotypic alterations associated with acquired gemcitabine resistance can change the cell-sorting behavior of pancreatic cancer cells, we generated co-culture spheroids of BxPC3 and BxGR-360C cells labeled with CellTracker Green or CellTracker Red prior to spheroid formation, and imaged the subpopulation distributions using multiphoton microscopy (MPM). As can be seen in Figs 6A and S4A, a well-mixed network of loosely connected cells forms in the attachment-free plates within 4 hours of seeding. Within 24 hours, we observe formation of spheroids with a striking preferential localization of the BxGR-360C cells to the outer shell (Figs 6A, 6B, S4A and S4B). To rule out any imaging or cell labelling bias, surface localization of RRM1 overexpressing cells was confirmed by cryosectioning and immunofluorescent staining (Fig 6C). Near-complete surface coverage of resistant cells was observed in spheroids with approximately 25% resistant cells (Fig 6D). To quantitatively show spheroid surface enrichment of BxGR-360C cells when co-cultured with BxPC3 cells, we analyzed images acquired on the IncuCyte S3 platform. Unlabeled and CellTracker Red labeled BxPC3 and BxGR-360C cells were cultured as mono- and co-culture spheroids. The radial fluorescent intensity profile was determined for each spheroid, averaged across 20 spheroids per condition and normalized to the spheroid center (Figs 6E and S5). We found that BxPC3/BxGR-360C co-culture spheroids displayed significant surface enrichment of BxGR-360C cells (Fig 6F). Additional spheroid co-culture replicates can be found in S4 and S6 Figs.

**Fig 7:**
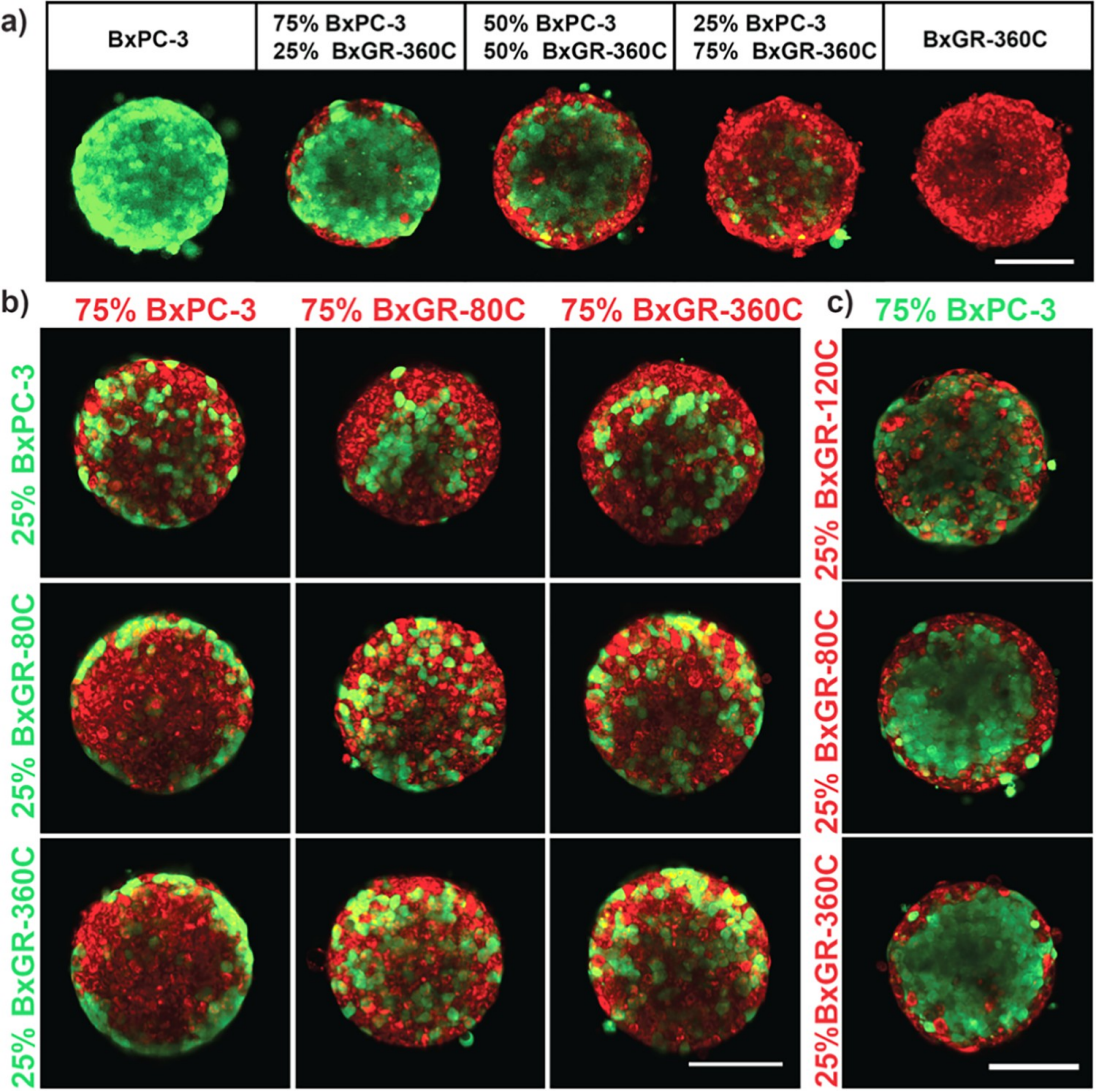
Multiphoton live-cell imaging of heterogeneous co-culture spheroids. a) Preferential surface localization of gemcitabine resistant cells (BxGR-360C, red) when cultured in spheroids with sensitive cell (BxPC3, green) observed across co-culture ratios. Scale bar 100 μm. b) Gemcitabine resistant cells BxGR-80C and BxGR-360C display preferential surface localization when co-cultured with drug-sensitive BxPC3 cells. No cell-sorting is observed in co-cultures of BxGR-80C and BxGR-360C cells or in spheroids consisting of only one cell type. Scale bar 100 μm. c) No cell-sorting is observed in co-cultures of BxGR-120C and BxPC-3 cells. A total of 2000 cells was seeded per well in all conditions, cells were labelled with CellTracker Green (green) or CellTracker Red (red) prior to seeding spheroids. Scale bar 100 μm. a-c) Shows representative images from a minimum of 3 experimental replicates.

We next sought to determine if the cell-sorting behavior was consistent across all gemcitabine resistant subclones. We found that both BxGR-80C and BxGR-360C preferentially localize to the spheroid surface when co-cultured with BxPC3, but that no cell-sorting occurs between BxGR-80C and BxGR-360C cells (Fig 7A and 7B). Furthermore, cell-sorting was observed between BxGR-120C and BxGR-80C/BxGR-360C, but not between BxGR-120C and BxPC3 (Fig 7C). This result was surprising as BxPC3 and BxGR-80C cells are almost indistinguishable morphologically, yet display cell-sorting, whereas BxGR-80C and BxGR-360C cells display starkly different morphologies, yet do not sort.

**Fig 8:**
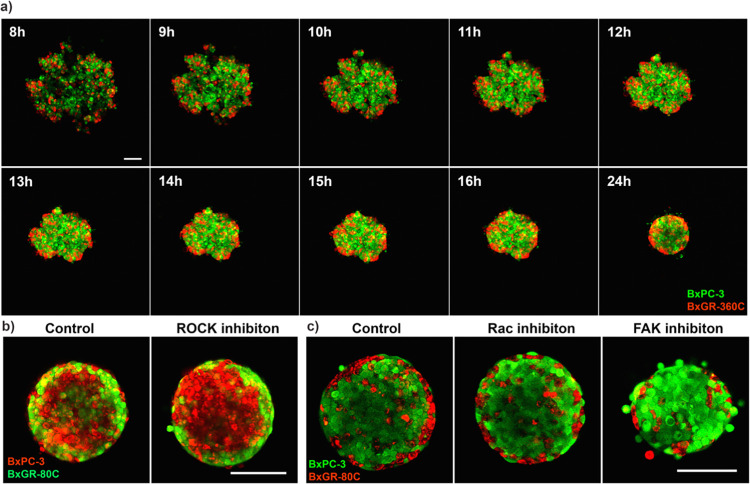
Time lapse imaging of co-culture spheroids. a) Live-cell time lapse multiphoton imaging of spheroid formation. 1500 gemcitabine sensitive BxPC-3 cells (green) were seeded with 500 gemcitabine-resistant BxGR-360C cells (red) in attachment free wells, time lapse imaging was started after 4 hours and spheroids were imaged every 60 minutes for 48 hours. Complete spheroid formation was observed after 24 hours. Scale bar 100 μm. b) Effect of ROCK-inhibition on cell-sorting in spheroids consisting of 1500 BxPC-3 (red) and 500 BxGR-80C (green) cells. Scale bar 100 μm. c) Effect of FAK- and Rac-inhibition on cell-sorting in spheroids consisting of 1500 BxPC-3 (green) and 500 BxGR-80C (red) cells. Scale bar 100 μm. a-c) Shows representative images from a minimum of 3 experimental replicates.

To look closer at the dynamics of spheroid formation, we also performed MPM time-lapse studies. These studies show that a loosely associated and well-mixed network of cells forms within 4 hours of seeding (Fig 6A) and that spheroid formation results from the relatively rapid contraction of this cellular network (Fig 8A). Looking in detail at the position of BxGR-360C cells when co-cultured with BxPC3 cells reveals that as the cellular network contracts, a majority of BxGR-360C cells are excluded from the spheroid core as it is forming. It is thus possible that cell-sorting results from the dysregulation of cell-cell junctions in BxGR-360C cells relative to BxPC3 cells. In some cases, a fraction of cells expected to preferentially localize to the surface would become trapped in the spheroid core, potentially due to “jamming’’ of the cells as the spheroid contracts. To investigate the relation of cell-cell junction strength to this pattern of observation, we generated spheroids in the presence of ROCK-inhibitor. ROCK activity is necessary for the maintenance, but not initial formation, of cell-cell junctions [47]. We observed that, in variety of cases, ROCK inhibition enhanced cell-sorting. Fig 8B shows fewer BxGR-80C cells being trapped in the core and fewer BxPC3 cells localizing to the outside of the BxGR-80C shell when treated with ROCK inhibitor. We hypothesize that ROCK inhibition weakens cell-cell junctions that form as the spheroid contracts, reducing the number of trapped cells. Additional replicates can be seen in S6A and S6B Fig. FAK- and Rac1-inhibition was found to interfere with cell-sorting in spheroids (Fig 8C). Rac-inhibition appears to decrease the fraction of BxGR-80C cells localizing to the surface. A similar effect was observed with FAK-inhibition, which in addition also resulted in the formation of smaller spheroids with a fraction of cells remaining unattached (Fig 8C).

### Gemcitabine-resistant cells exert a chemoprotective effect on spheroid co-cultures

As discussed previously, we hypothesize that the overexpression of RRM1 in BxGR-360C cells results in gemcitabine detoxification by supplying an excess of RRM1. By preferentially localizing to the surface of co-culture spheroids, it is possible that the presence of BxGR-360C cells may reduce the flux of gemcitabine into the spheroid core, and thus exert a chemoprotective effect on otherwise gemcitabine-sensitive cells. Surface localization of ABC drug transporter expressing cells in co-culture spheroids has previously been shown to markedly inhibit drug transport into the spheroid core [36]. To determine if such a protective effect is observable in our system, we quantified the induction of gemcitabine-induced cell death in spheroids consisting of varying ratios of resistant and sensitive cells.

**Fig 9:**
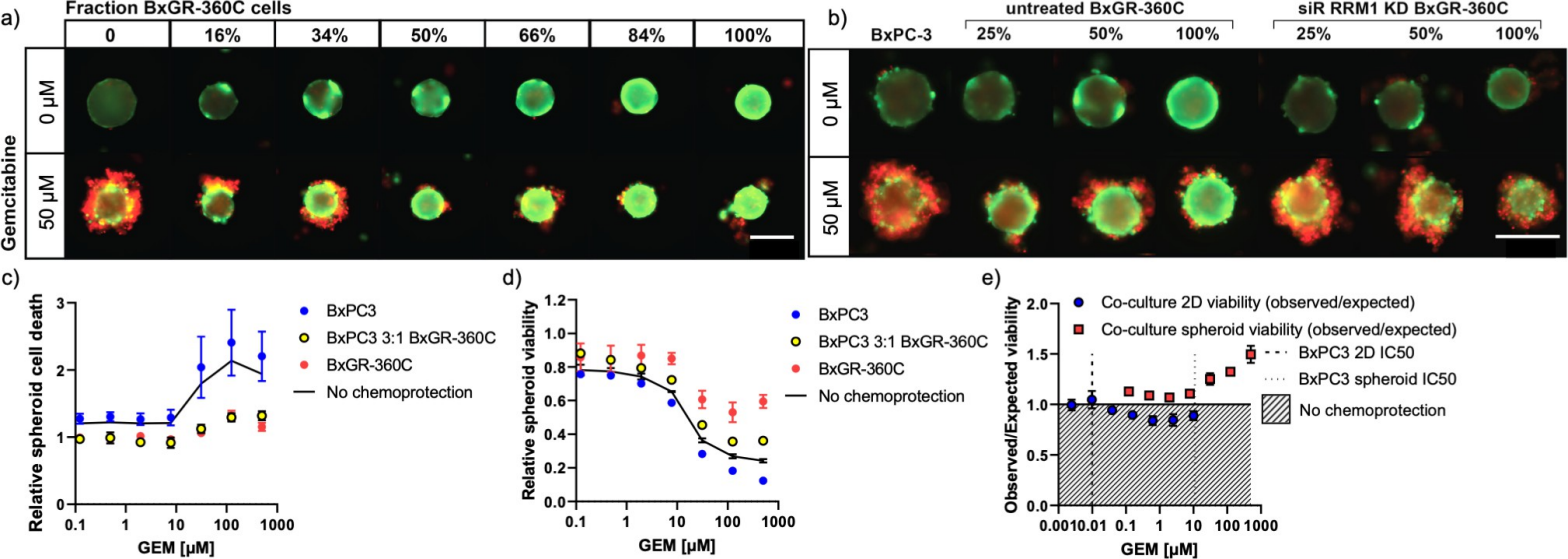
Characterization of gemcitabine dose-response in heterogeneous spheroids. a) Representative live/dead cell labeling images reveal a marked chemoprotective effect when a small fraction of gemcitabine-resistant BxGR-360C are co-cultured with drug-sensitive BxPC-3 cells. Calcein AM (green) labels live cells and propidium iodide (red) labels dead cells with compromised cell membranes. Scale bar 200 μm. b) Loss of protective effect when RRM1 is knocked-down in BxGR-360C cells prior to spheroid formation. Scale bar 200 μm. c) Quantitation of dead cells in response to gemcitabine incubation (6 days) in single-clone and heterogeneous spheroids as measured by PI uptake and automated image analysis. Black line indicates expected values for BxPC3 3:1 BxGR-360C co-culture spheroids assuming no protection (simple linear combination). Four independent experimental replicates. Error bars indicate SEM. d) Determination of spheroid viability in response to gemcitabine incubation (6 days) in single-clone and heterogeneous spheroids using CellTiter-Glo 3D across three independent experimental replicates. Black line indicates expected values for BxPC3 3:1 BxGR-360C co-culture spheroids assuming no protection (simple linear combination). Error bars indicate SEM. e) Ratio of observed and expected viability for heterogeneous 2D culture (MTT) and spheroids (CellTiter-Glo 3D) of BxPC3 cells co-cultured with BxGR-360C cells. The ratio was fixed to 3:1 (BxPC3:BxGR-360C) for all 2D and spheroid co-cultures. A total of 2000 cells were seeded per spheroid. Shaded area indicates observed viability lower than expected from simple linear combination of pure BxPC3 and BxGR-360C cultures (3:1 ratio), i.e. no protection. Dotted lines indicate BxPC3 gemcitabine IC50-values in 2D and spheroid culture. Error bars indicate SEM. See S7 Fig for 2D co-culture viability and cell death data. a-b) Shows representative images from a minimum of 3 experimental replicates.

Fig 9A reveals a marked protective effect from having a small fraction of BxGR-360C cells mixed into a population of BxPC3 cells in spheroid culture when exposed to 50 μM gemcitabine. This effect is readily observable when the fraction of resistant cells is 16% or higher (Fig 9A). Importantly, the protective effect was abolished when RRM1 was knocked down in the resistant cells prior to spheroid formation (Fig 9B). To quantify the protective effect, we determined the induction of cell death and loss of viability in response to gemcitabine using our previously optimized spheroid assays (Fig 5), as wells as in conventional 2D mono- and co-cultures (S7A and S7B Fig). Spheroids consisting of 75% BxPC3 cells and 25% BxGR-360C were indistinguishable from pure BxGR-360C spheroids in terms of cell death, as measured by PI uptake (Fig 9C), with minimal cell death observed across gemcitabine concentrations. Furthermore, co-culture spheroids showed markedly higher viability in response to gemcitabine relative to pure BxPC3 spheroids (Fig 9D). To determine if a quantitative protective effect in spheroid co-culture is detectable in our experiments, we compared the observed to the expected viability for spheroid and 2D co-cultures. We calculated an expected level of viability for the 2D and spheroid co-cultures based on the observed viabilities of the pure populations, and used this to normalize the observed co-culture viability. As can be see seen in Fig 9E, no chemoprotective effect is observed in 2D culture (observed/expected viability ≤ 1), however a marked protective effect is found in co-culture spheroids in the presence of gemcitabine (observed/expected viability > 1). This effect is particularly evident at gemcitabine concentrations above the observed IC50 value (approximately 11 μM) for pure BxPC3 spheroids. We hypothesize that the observed effect results from the depletion of gemcitabine in the spheroid microenvironment by the RRM1-overexpressing cells, in combination with limited mass transport of gemcitabine into the spheroid core. As such, these observations support the idea that a resistance mechanism expressed by a minority of cancer cells can result in chemoprotection of the cancer cell population as a whole.

### Gemcitabine-resistant cells drive collective migration

**Fig 10:**
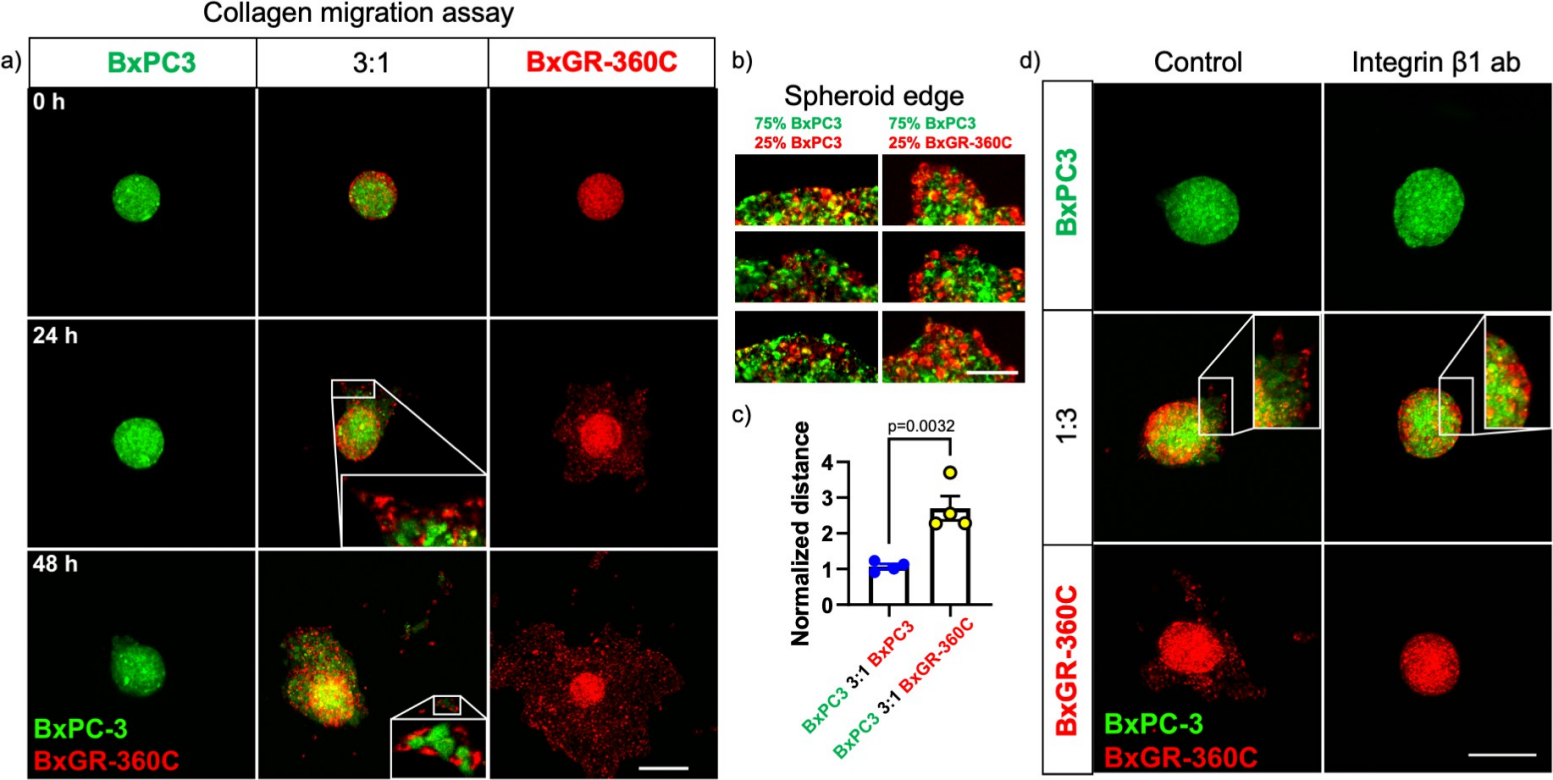
Collective migration. a) Collagen migration/invasion assay of BxPC3, BxGR-360C, and co-culture spheroids over 48 hours. BxPC3 (green) and BxGR-360C (red) cells labeled prior to seeding spheroids. Insets show the leading invasive edge (center, middle) and small cluster that has invaded completely (center, bottom). Maximum intensity projections of multiphoton microscopy z-stacks. Scale bar 200 μm. b) Magnified view of the invasive edge of 6 co-culture spheroids. BxPC3 (green) and BxGR-360C (red) cells labeled prior to seeding spheroids. Scale bar 100 μm. c) Normalized distance to spheroid center of the BxPC3 leading edge at the 48 hour collagen migration time-point. Four spheroids analyzed per spheroid type. The distance-to-center was measured and averaged for the three longest CTG-positive (BxPC3) invasive edges for each spheroid and normalized to the starting spheroid diameter. d) Inhibition of migration when incubating spheroids with integrin beta 1 antibody prior to spheroid collagen embedding. Scale bar 200 μm. a,b,d) Shows representative images from 3 experimental replicates.

As discussed above, the highly gemcitabine-resistant BxGR-360C cells display traits typical of an invasive phenotype. To investigate the 3D invasiveness of these cells, we developed a collagen migration/invasion assay for spheroids, based on previously described methods [20]. As can be seen in Fig 10A, we found that BxPC3 spheroids do not readily invade or migrate when embedded into type I collagen. This is consistent with previous studies showing that BxPC3 cells are relatively non-migratory and non-invasive, and predominantly migrate as cohesive cellular sheets [48]. Unlike BxPC3 cells, BxGR-360C spheroids showed rapid migration into the surrounding collagen, see Fig 10A. Although a small number of BxGR-360C cells were observed to migrate into collagen as single cells, migration predominantly occurred as collective migration of cellular sheets with retained cell-cell contacts. Previous studies have concluded that collective invasion is facilitated by a combination of invasive and epithelial traits [30]. Furthermore, invasive cells can enable invasion of less aggressive cells by acting as cellular trailblazers [18,20,30]. We hypothesized that the BxGR-360C cells may enable migration of the otherwise non-migratory BxPC3 cells. To test this, we generated co-culture spheroids, embedded the spheroids in a collagen I matrix and monitored migration over time. Co-culture enabled remarkable collective migration of the BxPC3 cells (Fig 10A). After 24 hours, the leading invasive edge in spheroid co-cultures consisted nearly entirely of BxGR-360C cells (inset in Fig 10A and 10B) and by 48 hours, small clusters of cells had completely broken off from the spheroids and invaded into the surrounding area. The perimeters of these clusters were preferentially made up of BxGR-360C cells, enclosing a core of BxPC3 cells (inset in Fig 10A). These observations are consistent with BxGR-360C cell enabling the migration of BxPC3 cells by forming an invasive edge. To quantify collective migration, we measured the distance between the spheroid center and the three furthest BxPC3 cell signatures (by fluorescent label) inside a continuous invasive cell sheet. This analysis confirmed increased BxPC3 migration in the presence of BxGR-360C cells (Fig 10C). To confirm that the observed migration is integrin-mediated, we blocked migration by activating integrin bonding with the TS2/16 integrin beta 1 antibody, resulting in complete inhibition, see Fig 10D. We hypothesize that the elevated lamellipodia formation observed in BxGR-360C cells increases the formation of focal adhesions with polymerized collagen fibers in the surrounding matrix, and through cell-cell junctions the BxGR-360C cells can exert force on their neighbors, facilitating collective migration.

In summary, our results show that acquired gemcitabine resistance has the potential to not only increase the invasive ability of the resistant subclone, but the invasiveness of the tumor cell population as a whole. We believe that the combination of an invasive phenotype with the retained ability to form cell-cell junctions is necessary for this effect to take place.

## Discussion

The interaction of tumor cell subpopulations is thought to in some cases give rise to collective behavior, potentially contributing to metastasis and chemoresistance. However, the extent to which this occurs in pathologically relevant settings has not been determined. We believe that the results described here constitute the first direct observation of the spontaneous emergence of collective chemoprotection and migration in closely related populations of cancer cells.

Taken together, the ability of chemoresistant cancer cells to exert a population-wide chemoprotective effect and enable migration/invasion of non-resistant cells has significant clinical implications. Specifically, if clusters of resistant and non-resistant cells can invade and intravasate, the clusters may be chemoprotected in circulation while maintaining proliferative potential. Clusters of circulating tumor cells have attracted intense attention in the last couple of years. These CTC clusters have been shown to consist of multiple cellular subclones [22] and to exhibit greatly increased metastatic potential over single CTCs [49]. Here, we demonstrate that the acquisition of chemoresistance has the potential to directly contribute to the migration of multiclonal cellular clusters.

Work by others have shown that highly invasive cancer cells can enable invasion of less aggressive cancer cells when co-cultured in spheroids. Specifically, a population of “invasion-competent’’ cancer cells can enable invasion of”invasion-incompetent’’ cells, giving rise to leader-follower dynamics [18,20,30]. These studies have predominately been performed in co-cultures of unrelated cancer cell lines, raising the question of whether the conditions necessary for facilitated collective migration can spontaneously arise within a tumor cell population. Our results show that collective migration can be enabled by cancer cells with aggressive traits gained through the acquisition of chemoresistance, and that these conditions are possible in subclones that are derived from the same parental cell population.

We chose to use BxPC3 cells for our study due to their pronounced epithelial phenotype and ability to form tight spheroids in culture. It should be noted that BxPC3 cells are KRAS wild-type and thus represent a minority (5–10%) of pancreatic cancer cases. Furthermore, KRAS wild-type pancreas cancers are through to arise from distinct oncogenic processes and potentially display unique therapeutic vulnerabilities, warranting closer study [50].

Previous studies have concluded that neither total cadherin expression (i.e. the sum of E-, N-, and P-cadherin), nor the resulting cellular adhesion force perfectly predicts cell-sorting behavior in cancer cell cultures [41]. Our results are in line with these observations, as we detect no significant changes in total E-cadherin and N-cadherin expression as a result of gemcitabine resistance, yet our cells display varying cell-sorting behaviors when co-cultured. Our results also show a marked dysregulation of E-cadherin localization in BxGR-360C cells, which may be involved in the cell-sorting behavior observed.

The work discussed here emphasizes that the aggressiveness of a cancer cell population cannot be determined by looking at the different cellular subpopulations separately. Here, we have demonstrated that complex collective cellular behaviors, such as cell-sorting, chemoprotection and collective migration, can emerge when populations of closely related cancer cells are cultured together. As such, the characteristics of a cancer tumor is not necessarily the sum of each of its cellular subpopulations, but rather emerges from the interaction of these subpopulations.

## Conclusion

The work discussed here emphasizes that the aggressiveness of a cancer cell population cannot be determined by looking at the different cellular subpopulations separately. Here, we have demonstrated that complex collective cellular behaviors, such as cell-sorting, chemoprotection and collective migration, can emerge when populations of closely related cancer cells are cultured together. As such, the characteristics of a cancer tumor is not necessarily the sum of each of its cellular subpopulations, but rather emerges from the interaction of these subpopulations.

## Supporting information

S1 Fig Characterization of gemcitabine-resistant subclones. a) Gemcitabine dose-response MTT assay in gemcitabine-resistant subclones derived from the human pancreatic cancer cell line BxPC3. Solid lines indicate three-parameter non-linear dose-response curves fitted to the aggregated data across all experiments. Error bars indicate standard deviation (SD). b) Confluency data generated through automated image analysis on the IncuCyte S3 platform. Solid lines indicated logistic growth functions fitted to the aggregated subclone data across all experiments. Error bars indicate SD. Three independent experimental replicates were performed for each subclone. c-d) Western blot and estimation of siRNA-mediated RRM1 knock-down in gemcitabine resistant BxGR-360C cells relative to control siRNA transfection. Data represents two independent transfections. e) Gemcitabine dose-response MTT assay of gemcitabine resistant BxGR-360C cells following siRNA-mediated RRM1 knock-down and control siRNA transfection. Three-parameter non-linear dose-response curves fitted to the aggregated data across all experiments. f) Quantification of gemcitabine IC50 values following siRNA-mediated knock-down in BxGR-360C cells. IC50 values determined from three-parameter non-linear dose-response curves fitted to three independent experimental replicates. Statistical significance determined using two-tailed Student’s t-test. Error-bars indicate SEM. g) Immunofluorescent staining of E-cadherin, vimentin and N-cadherin in BxPC3 and BxGR-360C cells. Scale bar 50 μm. h) Fluorescent images showing phalliodin (F-actin) and E-cadherin staining in BxPC3, BxGR-80C and BxGR-360C cells. BxGR-360C cells display remodeled actin cytoskeleton, including increased lamellipodia formation (top inset), and decreased E-cadherin localization to cell-cell junctions (bottom inset), scale bar 50 μm. i) Image analysis of E-cadherin enrichment at cell-cell junctions in BxGR-360C cells relative to BxPC3 cells. E-cadherin enrichment at cell-cell junctions was determined by calculating the ratio of the mean E-cadherin intensity proximal to the cellular edge and the mean E-cadherin intensity in the rest of the cell using CellProfiler, see S2 Fig for analysis pipeline. The displayed values indicate the degree of enrichment above mean cytoplasmic intensity. Three fields-of-view were analyzed per subclone, representing a minimum of 160 cells per field-of view. Statistical significance was determined with two-tailed Student’s t-test.
(TIF)

S2 Fig CellProfiler pipeline for cell-cell junction enrichment of E-cadherin. Cell nuclei are identified as primary objects using their DAPI signature. Cell outlines are identified by propagation, using E-cadherin staining and with nuclei as seeds. Expanded and shrunken cell outlines are defined by expanding and shrinking the cell outline by 2 and 7 pixels, respectively. The cell edge was defined as the area resulting from the subtraction of the shrunken from the expanded outline. The integrated mean E-cadherin intensity is determined and averaged for the edge regions and shrunken cytoplasm for each cell.
(TIF)

S3 Fig Estimation of cell death in spheroid cultures. a) Following 6 days of culture with various concentrations of gemcitabine, propidium iodide (PI) is added to visualize dead cells (cells with loss of cell membrane integrity). Spheroids are imaged on the IncuCyte S3 platform. Scale bar 100 μm. b) Spheroid cell death is estimated by calculating the integrated PI intensity across the spheroid area for each well and normalizing to untreated controls.
(TIF)

S4 Fig Additional co-culture spheroid images. a) Replicates of BxPC3 (green) and BxGR-360C (red) co-culture spheroids (3:1 ratio) 4 and 24 hours after seeding. 2000 total cells per spheroid. Scale bar 100 μm. b) Replicates of BxPC3 (red) and BxGR-360C (green) co-culture spheroids (3:1 ratio). 2000 cells per spheroid. Scale bar 100 μm. c) Comparison of confocal (single-photon) and multiphoton imaging microscopy (MPM). Note improved imaging of spheroid center with MPM. Scale bar 100 μm.
(TIF)

S5 Fig Determination of cell sorting in spheroids. a) Co-culture spheroids with CellTracker Green and CellTracker Red labeled cells are imaged on the IncuCyte S3 platform. b-d) The radial distribution profile of CellTracker Red intensity is determined for the extracted images using the Radial Profile plugin in ImageJ. e) The intensity profiles are averaged across spheroids of the same spheroid type and normalized to the intensity in the spheroid center. The average spheroid radius for each spheroid type is defined as the distance from the center that encloses 95% of the total intensity for each spheroid type, and the center intensities are normalized to 1.
(TIF)

S6 Fig Additional co-culture spheroid images. a) Replicates of BxPC3 (red) and BxGR-80C (green) co-culture spheroids (1:1 and 3:1 ratio) with and without ROCK inhibitor. 2000 cells per spheroid. Scale bar 100 μm. b) Replicates of BxPC3 (red) and BxGR-360C (green) co-culture spheroids (3:1 ratio) with and without ROCK inhibitor. 2000 cells per spheroid. Scale bar 100 μm.
(TIF)

S7 Fig Characterization of gemcitabine dose-response in BxPC3 and BxGR-360C 2D mono- and co-culture. a) Gemcitabine dose-response MTT assay for BxPC3 and BxGR-360C 2D mono- and co-culture (3:1, BxPC3:BxGR-360C culture ratio). Solid black line indicates expected co-culture values assuming no protection (simple linear combination). Four independent experimental replicates were performed per culture condition. Error bars indicate standard deviation. b) Normalized cell death as a function of gemcitabine dose for BxPC3 and BxGR-360C 2D mono- and co-culture (3:1, BxPC3:BxGR-360C culture ratio) as measured by total CytoTox Red positive area per well determined in the IncuCyte S3 system, normalized to well viability (MTT) and untreated controls. Three independent experimental replicates were performed per culture condition. Solid black line indicates expected co-culture values assuming no protection (simple linear combination). Error bars indicate standard deviation.
(TIF)

S1 Table Differential Gene Expression, GSEA analysis and gene expression values. (XLSX)

S1 Raw images Original western blots of gemcitabine resistant subclones. (PDF)

S2 Raw images (TIF)
