# Supplementary figures and images for: Acquired chemoresistance drives spatial heterogeneity, chemoprotection and collective migration in pancreatic tumor spheroids
Source: PLoS One. 2022 May 26;17(5):e0267882. doi: 10.1371/journal.pone.0267882 (PMC9135276; doi:10.1371/journal.pone.0267882)

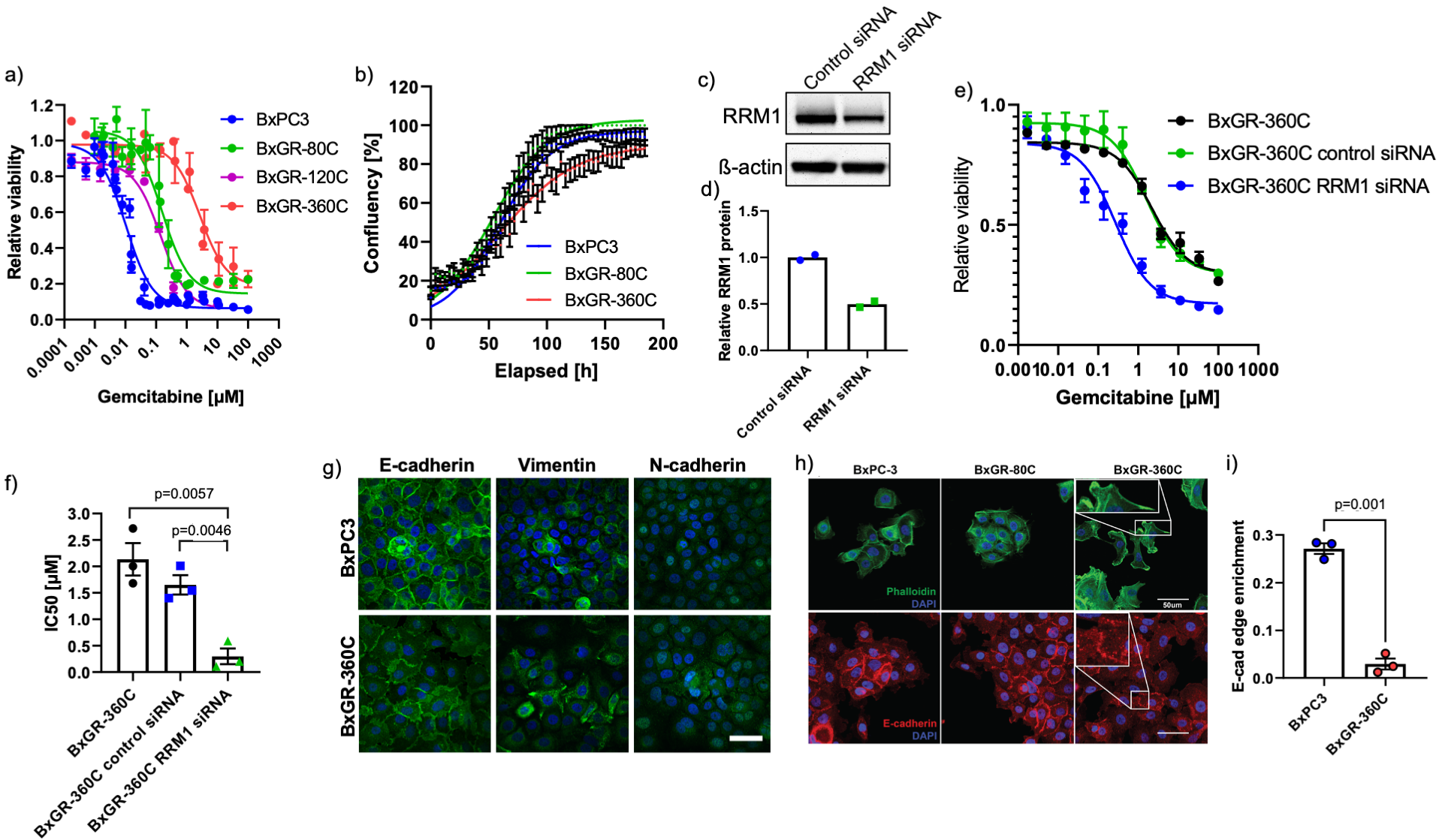

Supplement: S1 Fig — a) Gemcitabine dose-response MTT assay in gemcitabine-resistant subclones derived from the human pancreatic cancer cell line BxPC3. Solid lines indicate three-parameter non-linear dose-response curves fitted to the aggregated data across all experiments. Error bars indicate standard deviation (SD). b) Confluency data generated through automated image analysis on the IncuCyte S3 platform. Solid lines indicated logistic growth functions fitted to the aggregated subclone data across all experiments. Error bars indicate SD. Three independent experimental replicates were performed for each subclone. c-d) Western blot and estimation of siRNA-mediated RRM1 knock-down in gemcitabine resistant BxGR-360C cells relative to control siRNA transfection. Data represents two independent transfections. e) Gemcitabine dose-response MTT assay of gemcitabine resistant BxGR-360C cells following siRNA-mediated RRM1 knock-down and control siRNA transfection. Three-parameter non-linear dose-response curves fitted to the aggregated data across all experiments. f) Quantification of gemcitabine IC50 values following siRNA-mediated knock-down in BxGR-360C cells. IC50 values determined from three-parameter non-linear dose-response curves fitted to three independent experimental replicates. Statistical significance determined using two-tailed Student’s t-test. Error-bars indicate SEM. g) Immunofluorescent staining of E-cadherin, vimentin and N-cadherin in BxPC3 and BxGR-360C cells. Scale bar 50 μm. h) Fluorescent images showing phalliodin (F-actin) and E-cadherin staining in BxPC3, BxGR-80C and BxGR-360C cells. BxGR-360C cells display remodeled actin cytoskeleton, including increased lamellipodia formation (top inset), and decreased E-cadherin localization to cell-cell junctions (bottom inset), scale bar 50 μm. i) Image analysis of E-cadherin enrichment at cell-cell junctions in BxGR-360C cells relative to BxPC3 cells. E-cadherin enrichment at cell-cell junctions was determined by calculating th [file pone.0267882.s001.tif]

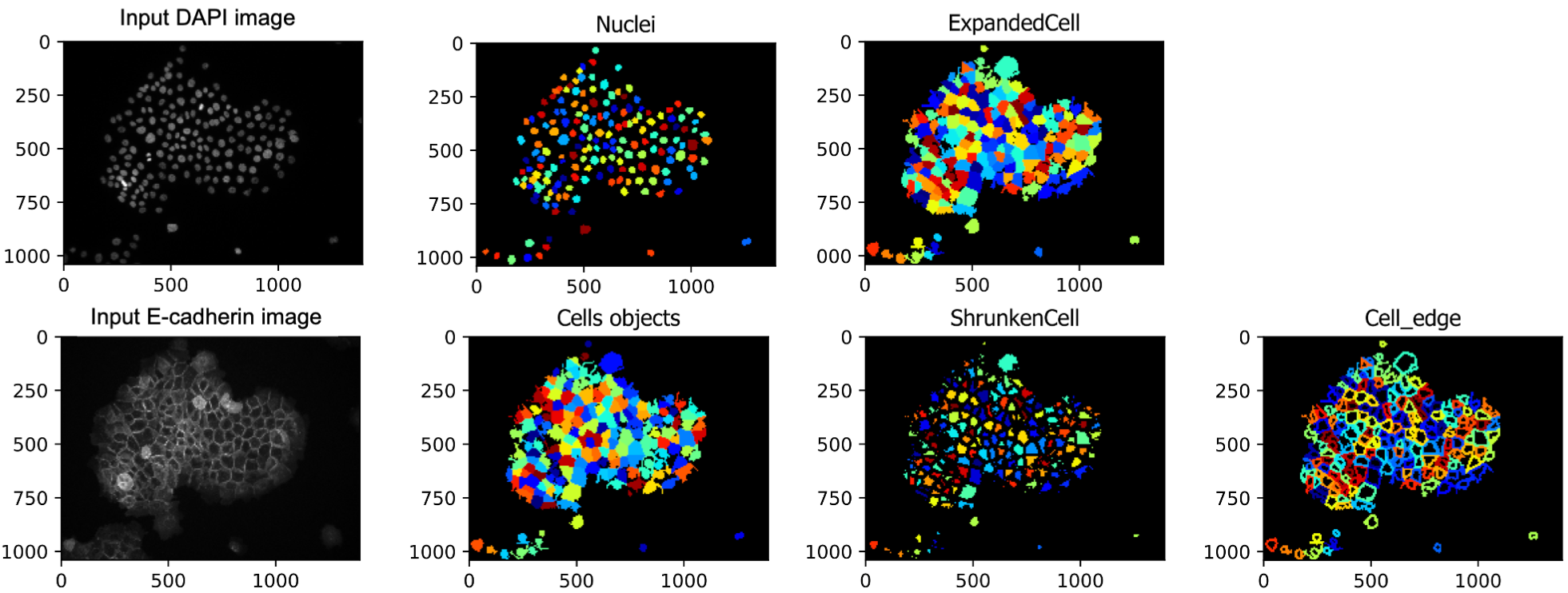

Supplement: S2 Fig — Cell nuclei are identified as primary objects using their DAPI signature. Cell outlines are identified by propagation, using E-cadherin staining and with nuclei as seeds. Expanded and shrunken cell outlines are defined by expanding and shrinking the cell outline by 2 and 7 pixels, respectively. The cell edge was defined as the area resulting from the subtraction of the shrunken from the expanded outline. The integrated mean E-cadherin intensity is determined and averaged for the edge regions and shrunken cytoplasm for each cell. (TIF) [file pone.0267882.s002.tif]

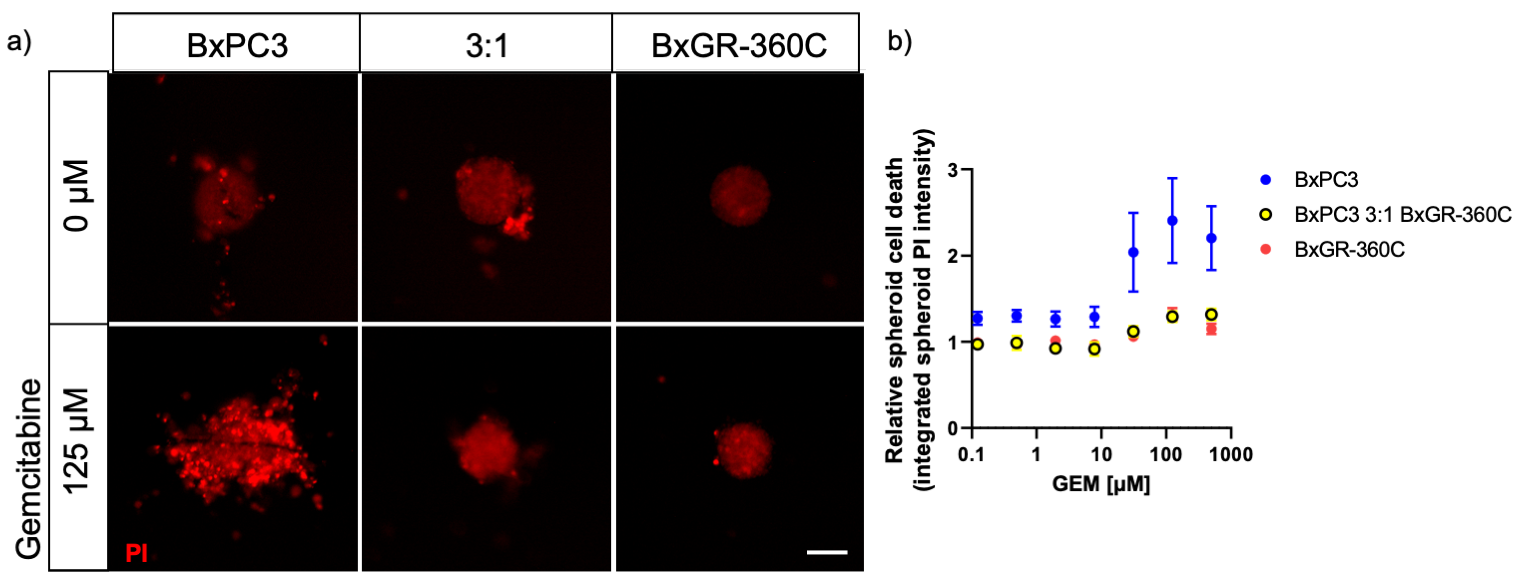

Supplement: S3 Fig — a) Following 6 days of culture with various concentrations of gemcitabine, propidium iodide (PI) is added to visualize dead cells (cells with loss of cell membrane integrity). Spheroids are imaged on the IncuCyte S3 platform. Scale bar 100 μm. b) Spheroid cell death is estimated by calculating the integrated PI intensity across the spheroid area for each well and normalizing to untreated controls. (TIF) [file pone.0267882.s003.tif]

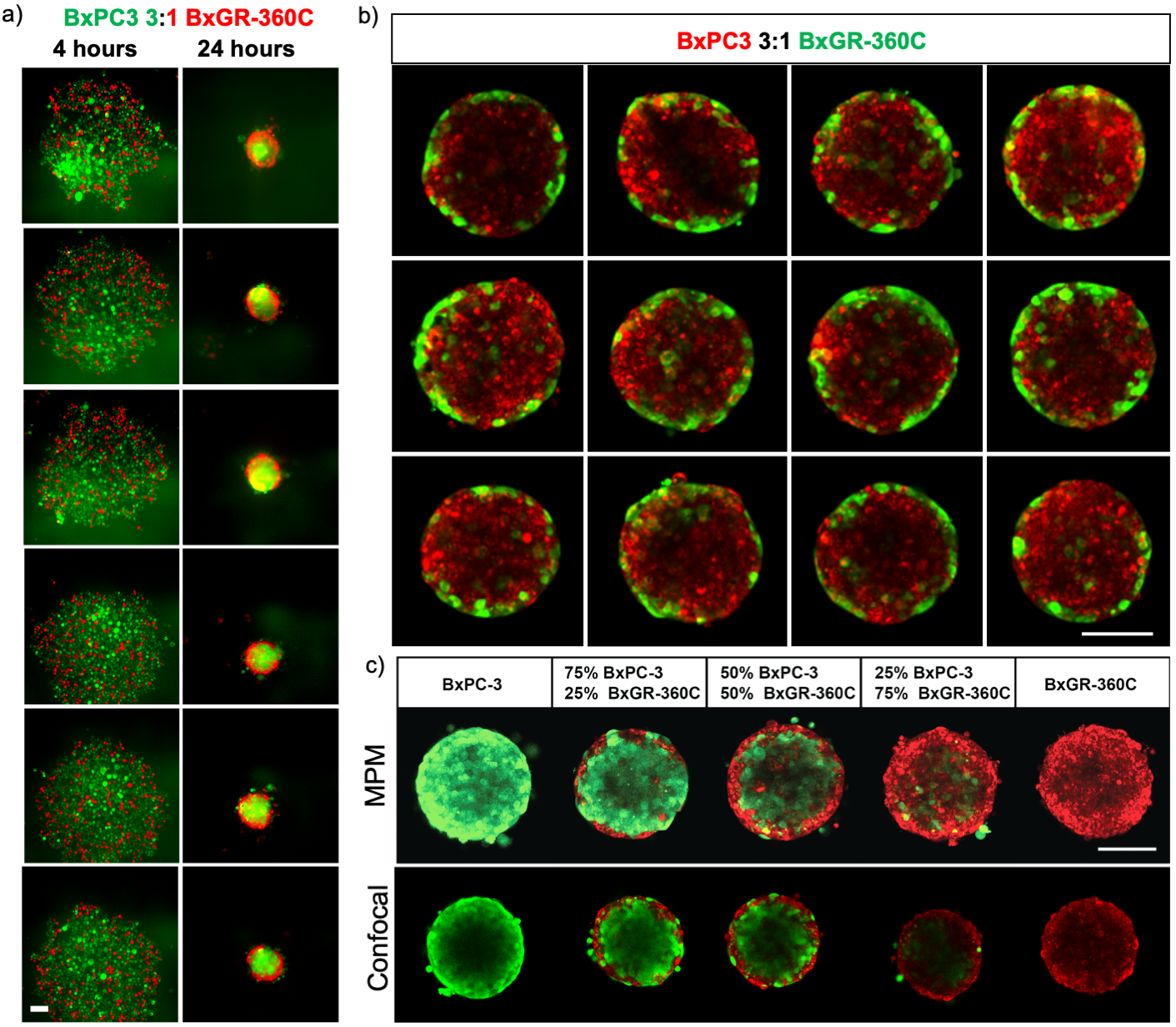

Supplement: S4 Fig — a) Replicates of BxPC3 (green) and BxGR-360C (red) co-culture spheroids (3:1 ratio) 4 and 24 hours after seeding. 2000 total cells per spheroid. Scale bar 100 μm. b) Replicates of BxPC3 (red) and BxGR-360C (green) co-culture spheroids (3:1 ratio). 2000 cells per spheroid. Scale bar 100 μm. c) Comparison of confocal (single-photon) and multiphoton imaging microscopy (MPM). Note improved imaging of spheroid center with MPM. Scale bar 100 μm. (TIF) [file pone.0267882.s004.tif]

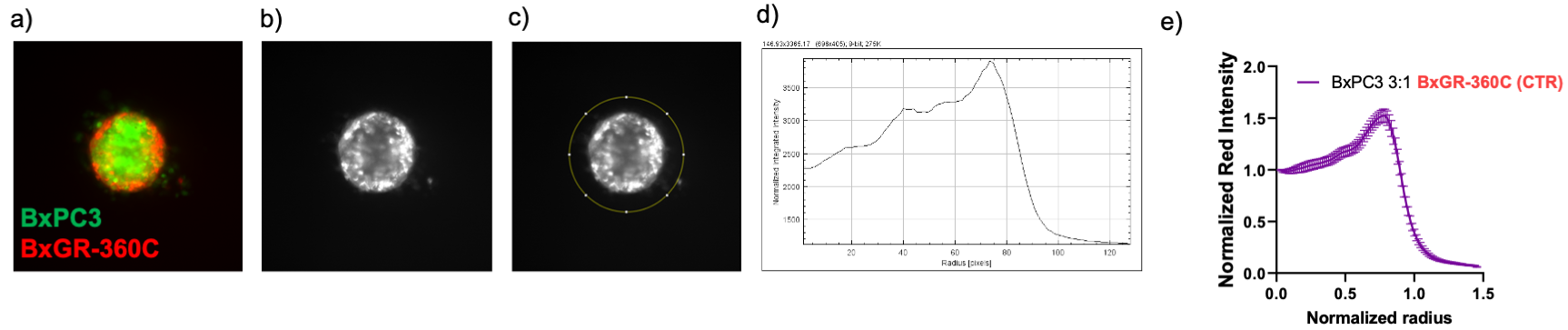

Supplement: S5 Fig — a) Co-culture spheroids with CellTracker Green and CellTracker Red labeled cells are imaged on the IncuCyte S3 platform. b-d) The radial distribution profile of CellTracker Red intensity is determined for the extracted images using the Radial Profile plugin in ImageJ. e) The intensity profiles are averaged across spheroids of the same spheroid type and normalized to the intensity in the spheroid center. The average spheroid radius for each spheroid type is defined as the distance from the center that encloses 95% of the total intensity for each spheroid type, and the center intensities are normalized to 1. (TIF) [file pone.0267882.s005.tif]

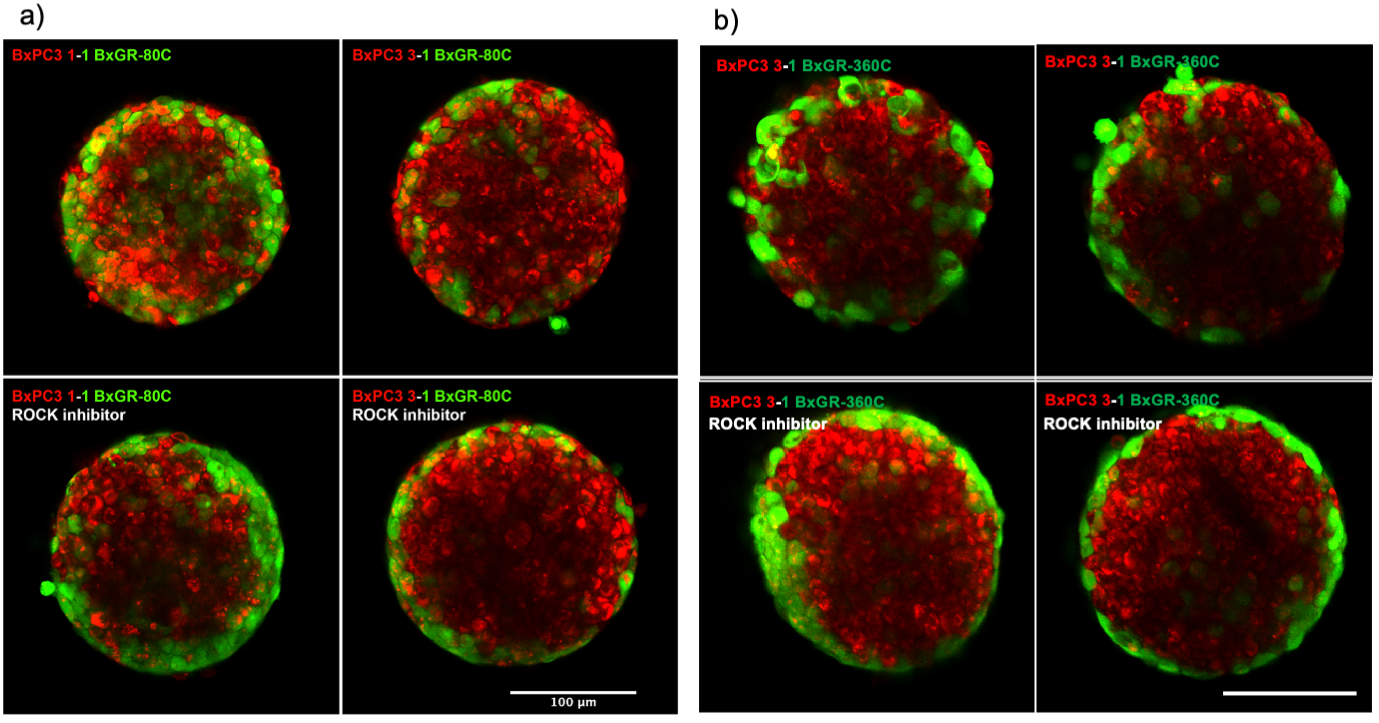

Supplement: S6 Fig — a) Replicates of BxPC3 (red) and BxGR-80C (green) co-culture spheroids (1:1 and 3:1 ratio) with and without ROCK inhibitor. 2000 cells per spheroid. Scale bar 100 μm. b) Replicates of BxPC3 (red) and BxGR-360C (green) co-culture spheroids (3:1 ratio) with and without ROCK inhibitor. 2000 cells per spheroid. Scale bar 100 μm. (TIF) [file pone.0267882.s006.tif]

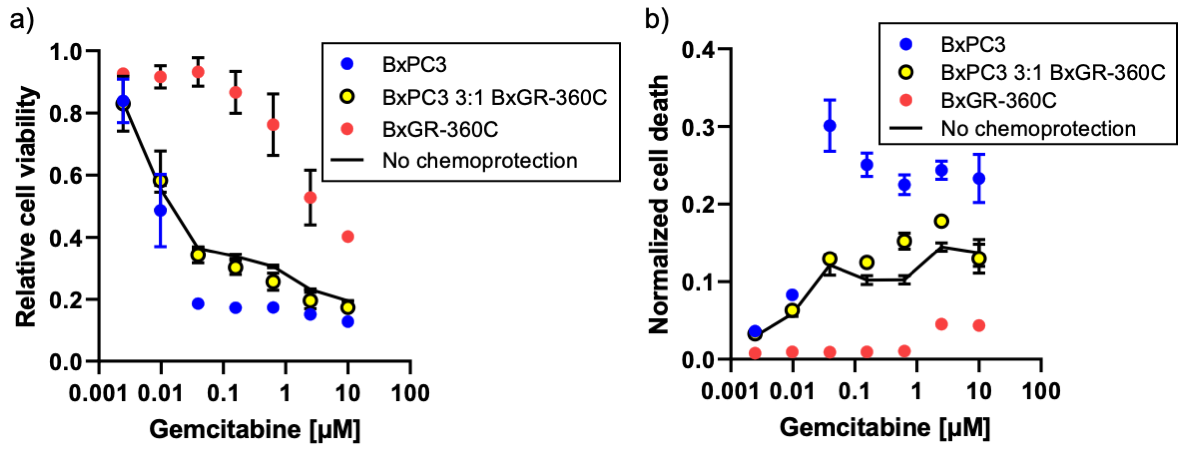

Supplement: S7 Fig — a) Gemcitabine dose-response MTT assay for BxPC3 and BxGR-360C 2D mono- and co-culture (3:1, BxPC3:BxGR-360C culture ratio). Solid black line indicates expected co-culture values assuming no protection (simple linear combination). Four independent experimental replicates were performed per culture condition. Error bars indicate standard deviation. b) Normalized cell death as a function of gemcitabine dose for BxPC3 and BxGR-360C 2D mono- and co-culture (3:1, BxPC3:BxGR-360C culture ratio) as measured by total CytoTox Red positive area per well determined in the IncuCyte S3 system, normalized to well viability (MTT) and untreated controls. Three independent experimental replicates were performed per culture condition. Solid black line indicates expected co-culture values assuming no protection (simple linear combination). Error bars indicate standard deviation. (TIF) [file pone.0267882.s007.tif]

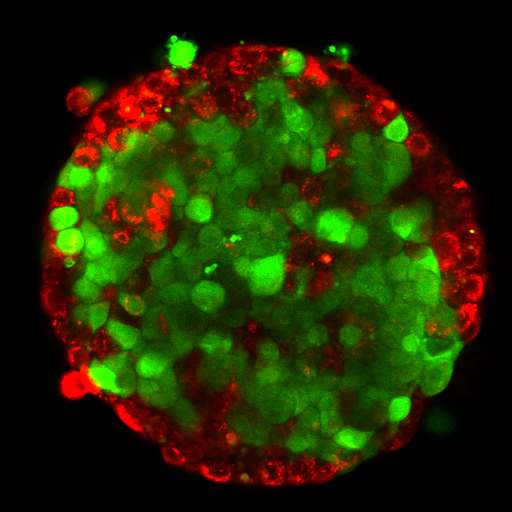

Supplement: S2 Raw images — (TIF) [file pone.0267882.s010.tif]
