# Supplementary material for: Acquired chemoresistance drives spatial heterogeneity, chemoprotection and collective migration in pancreatic tumor spheroids
Source: PLoS One. 2022 May 26;17(5):e0267882. doi: 10.1371/journal.pone.0267882 (PMC9135276; doi:10.1371/journal.pone.0267882)
Supplement: S1 Raw images — (PDF) [file pone.0267882.s009.pdf]

S1_raw_images.pdf

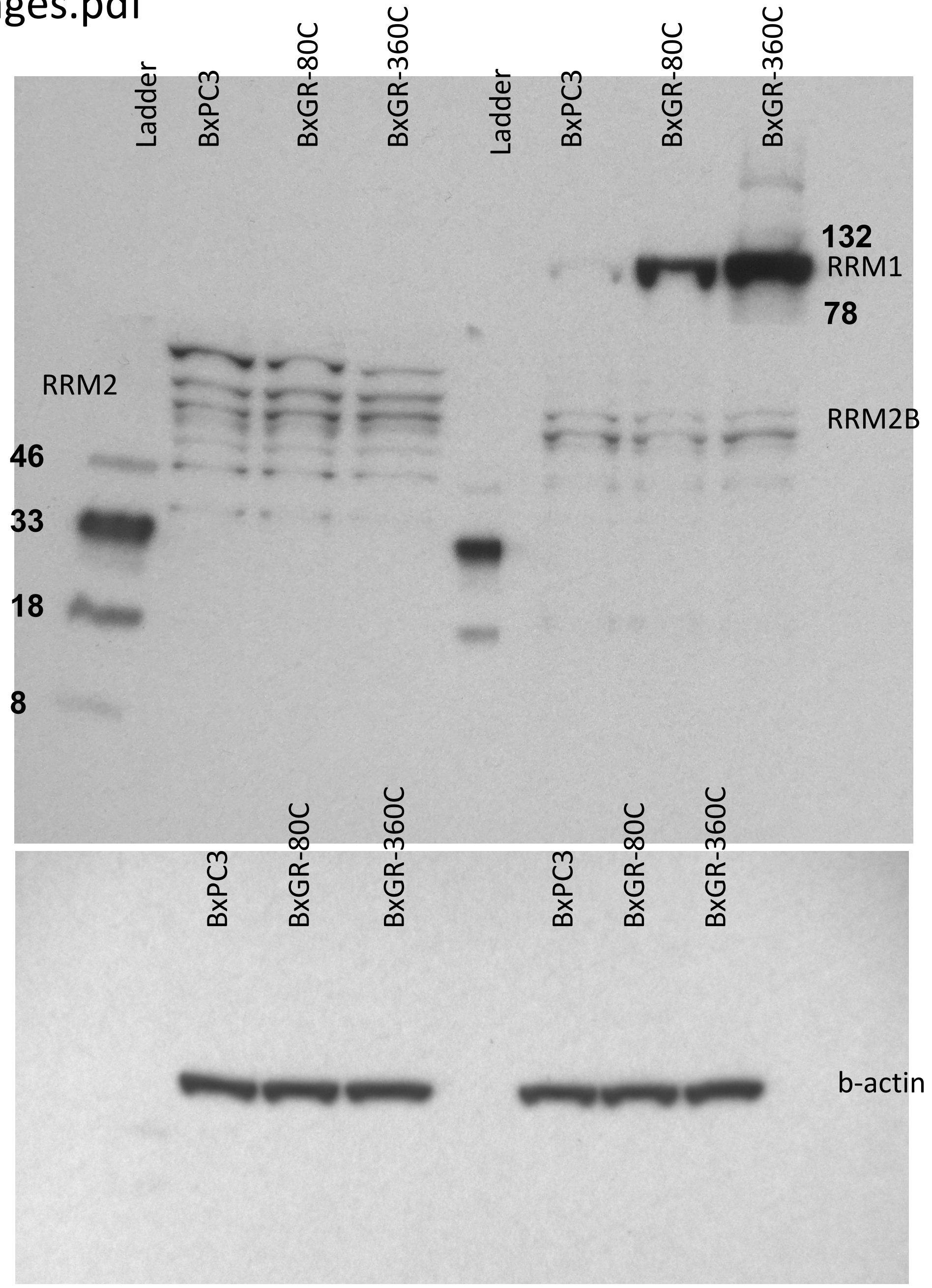

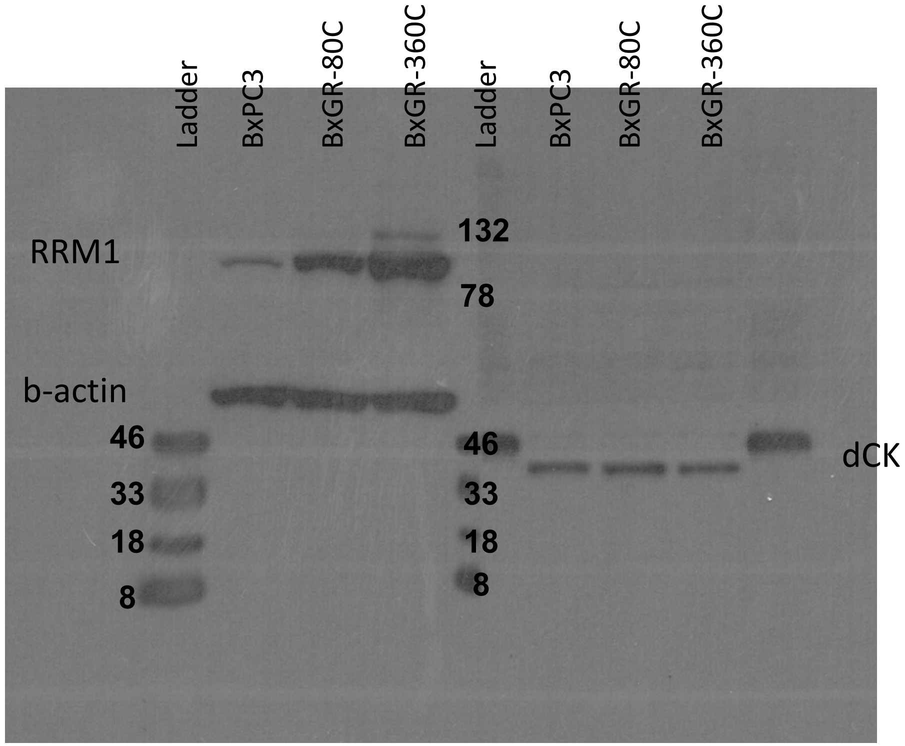

Supporting information. Original western blots of gemcitabine resistant subclones.

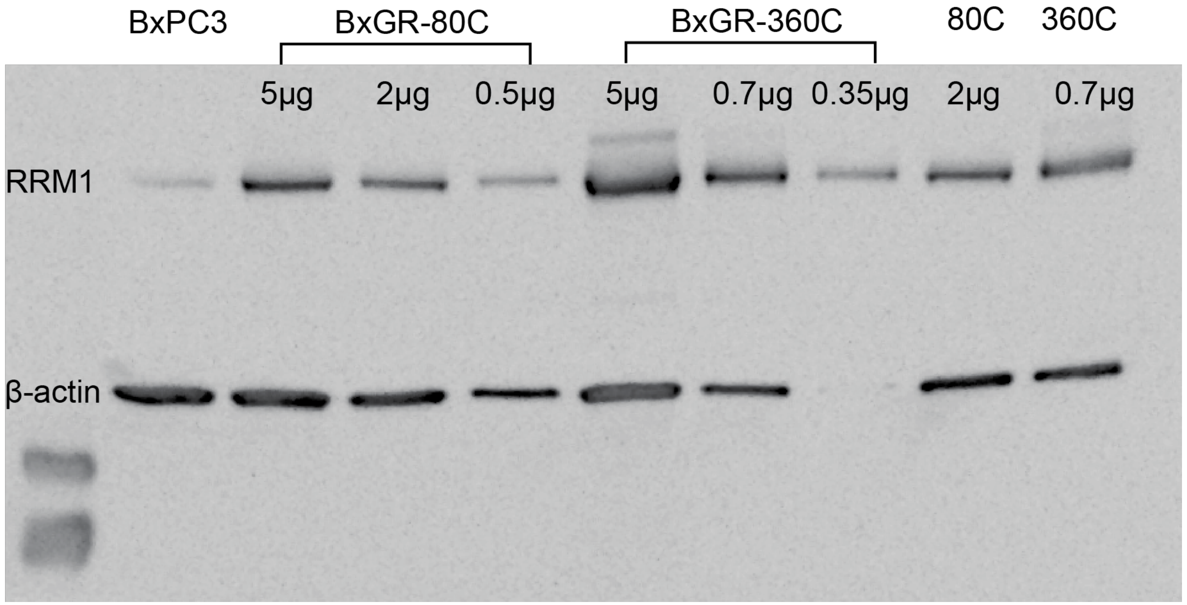

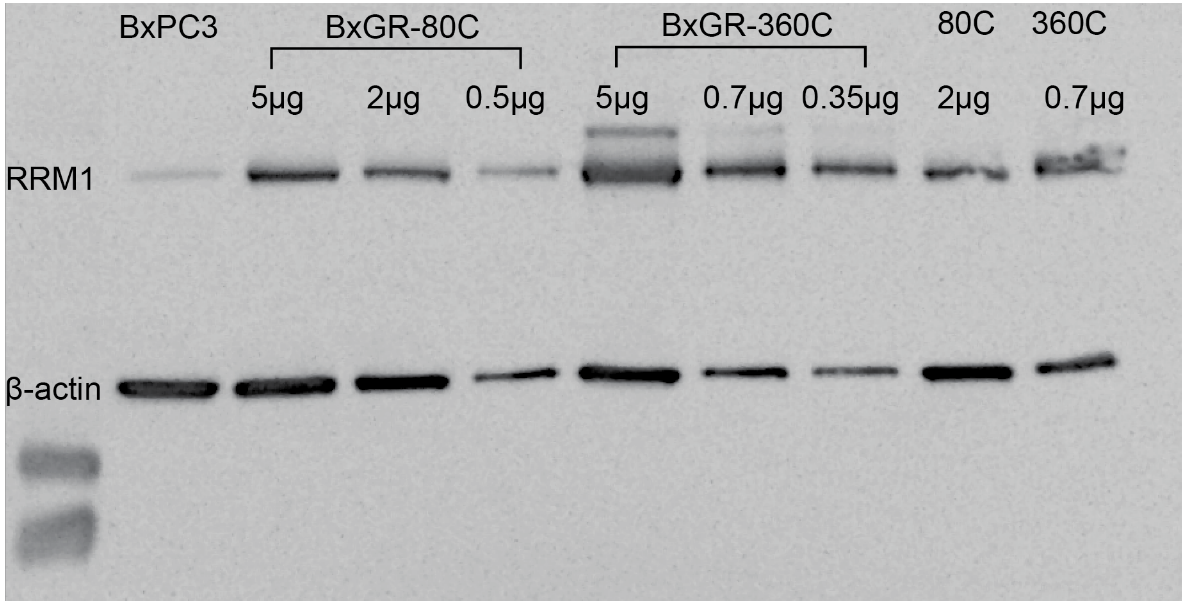

Supporting information. Original western blots of gemcitabine resistant subclones.

WB gemcitabine resistant clones

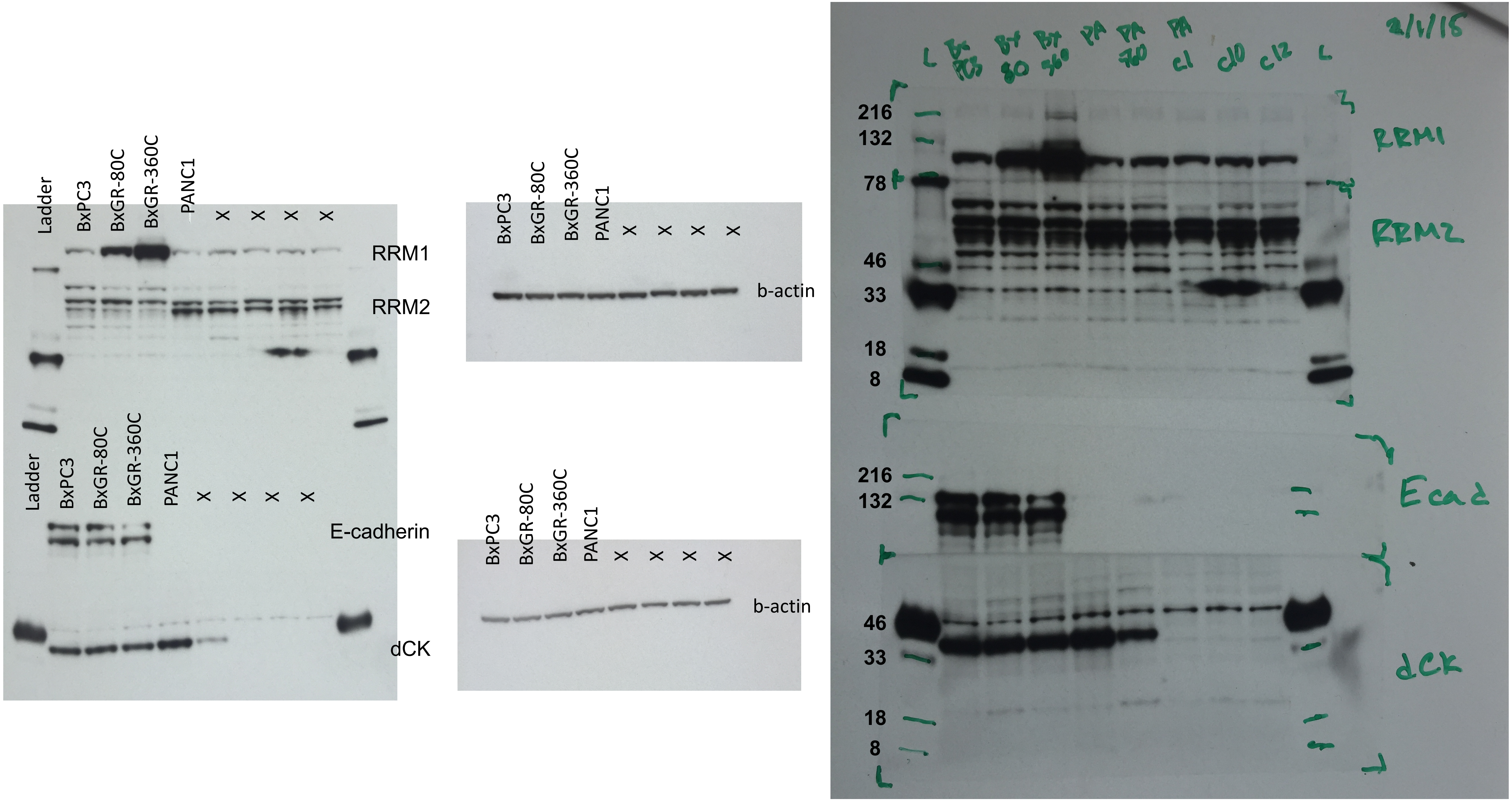

Supporting information. Original western blots of gemcitabine resistant subclones.

WB gemcitabine resistant clones

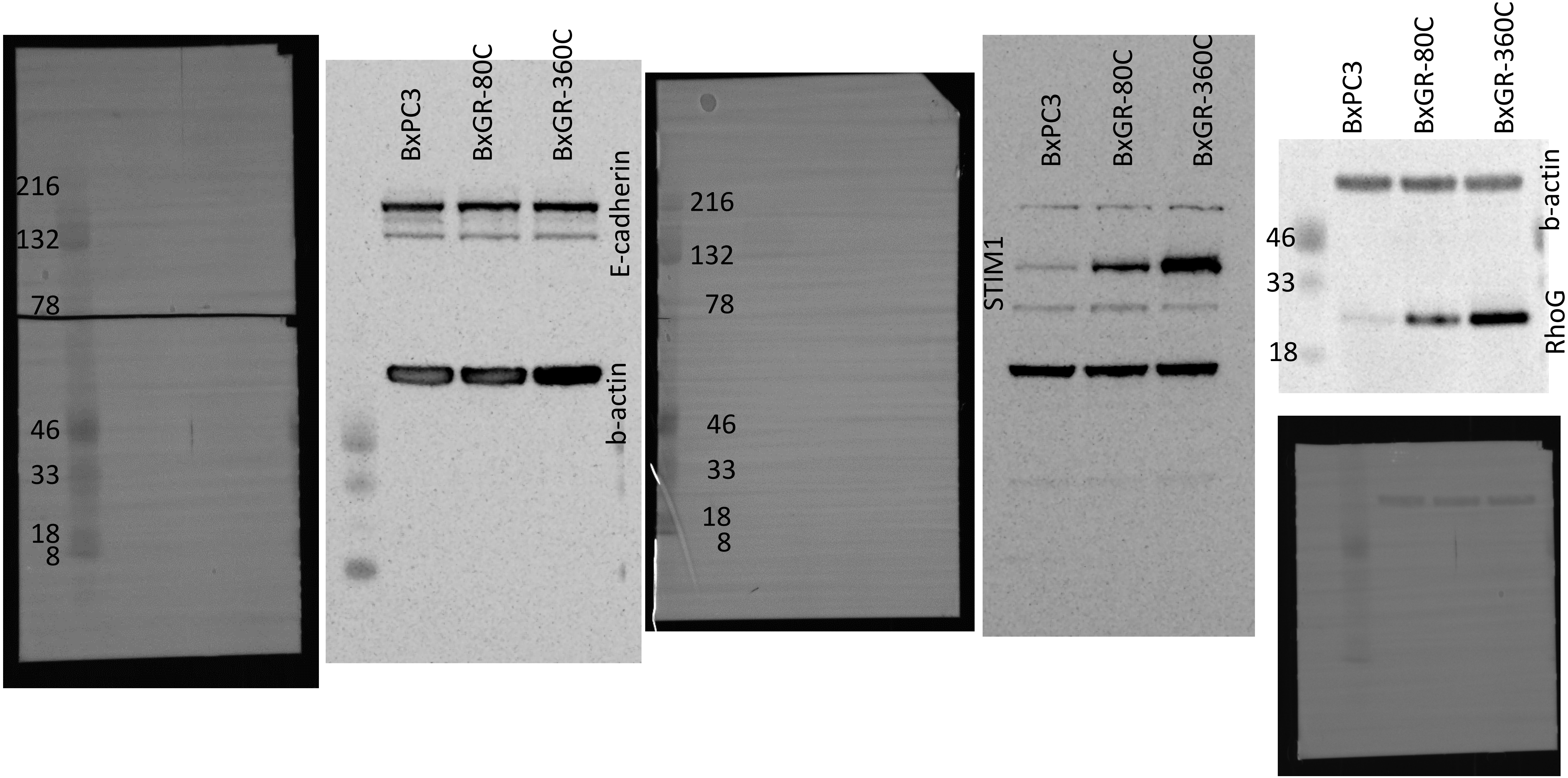

Supporting information. Original western blots of gemcitabine resistant subclones.

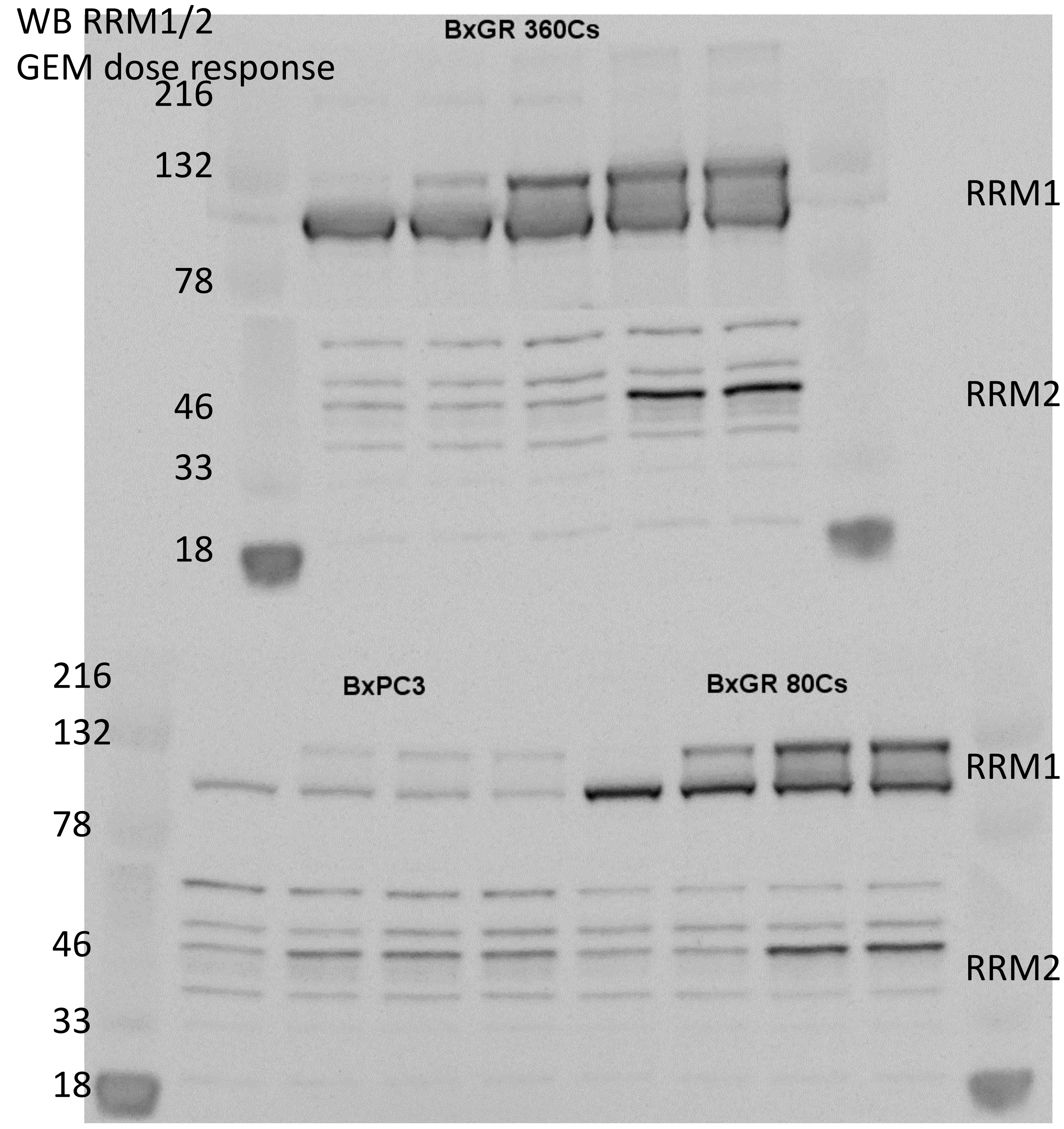

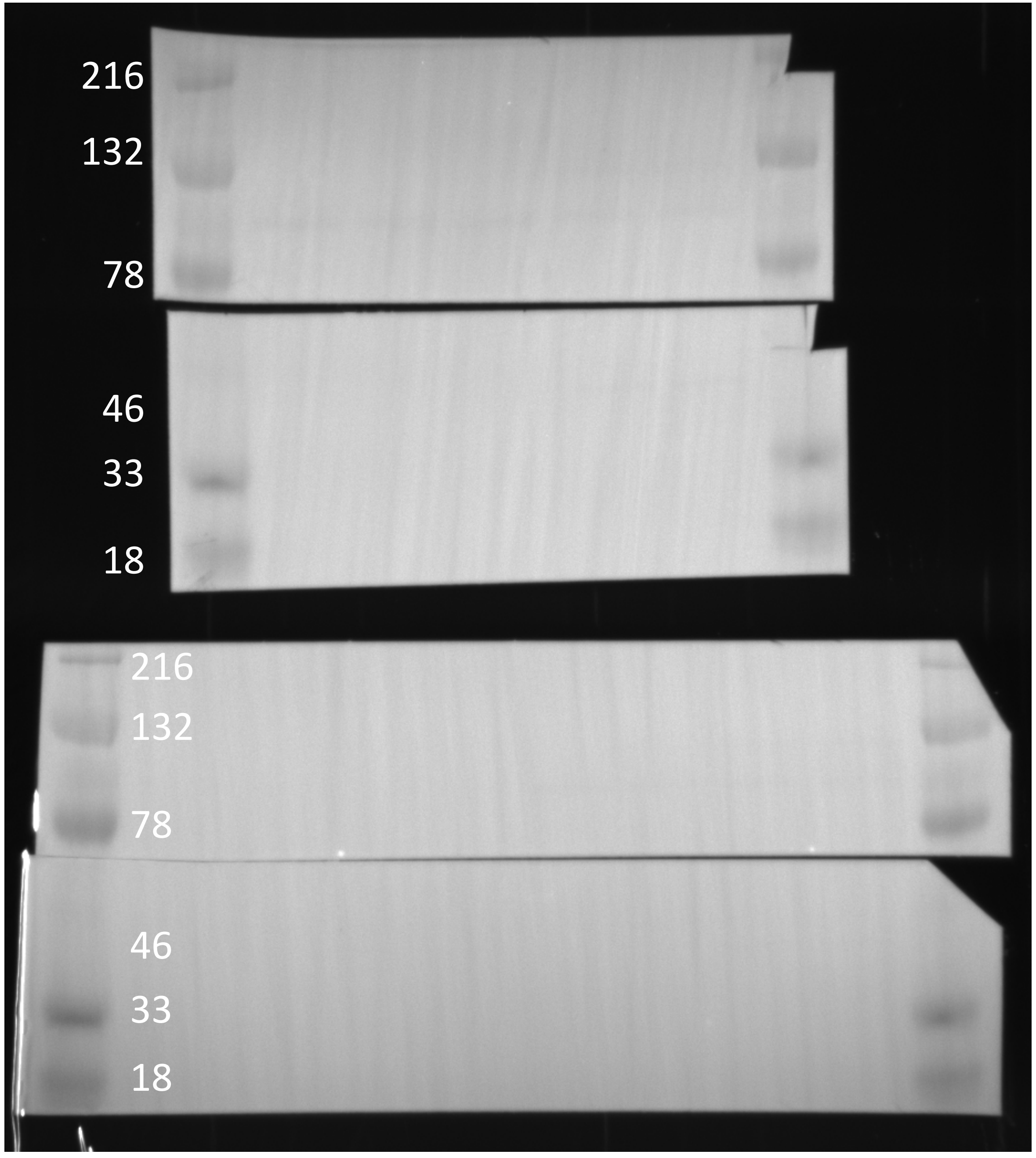

Supporting information. Original western blots of gemcitabine resistant subclones.

RRM1 siRNA KO blots

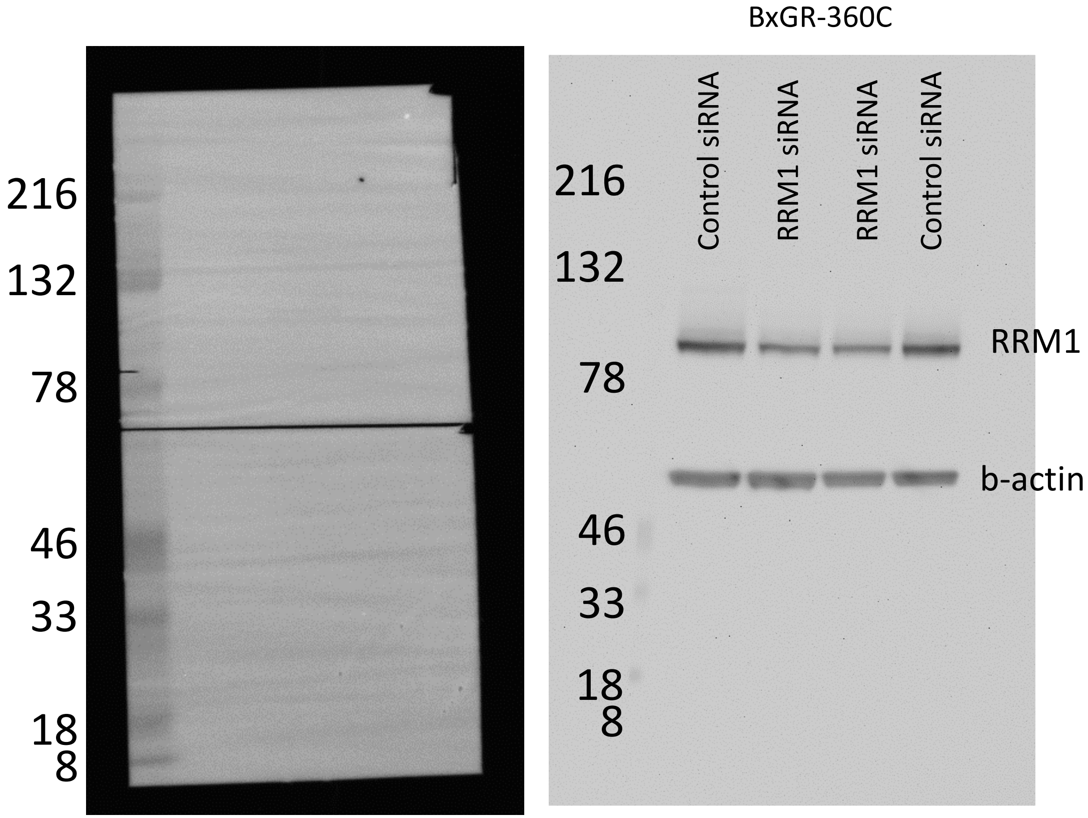

Supporting information. Original western blots of gemcitabine resistant subclones.

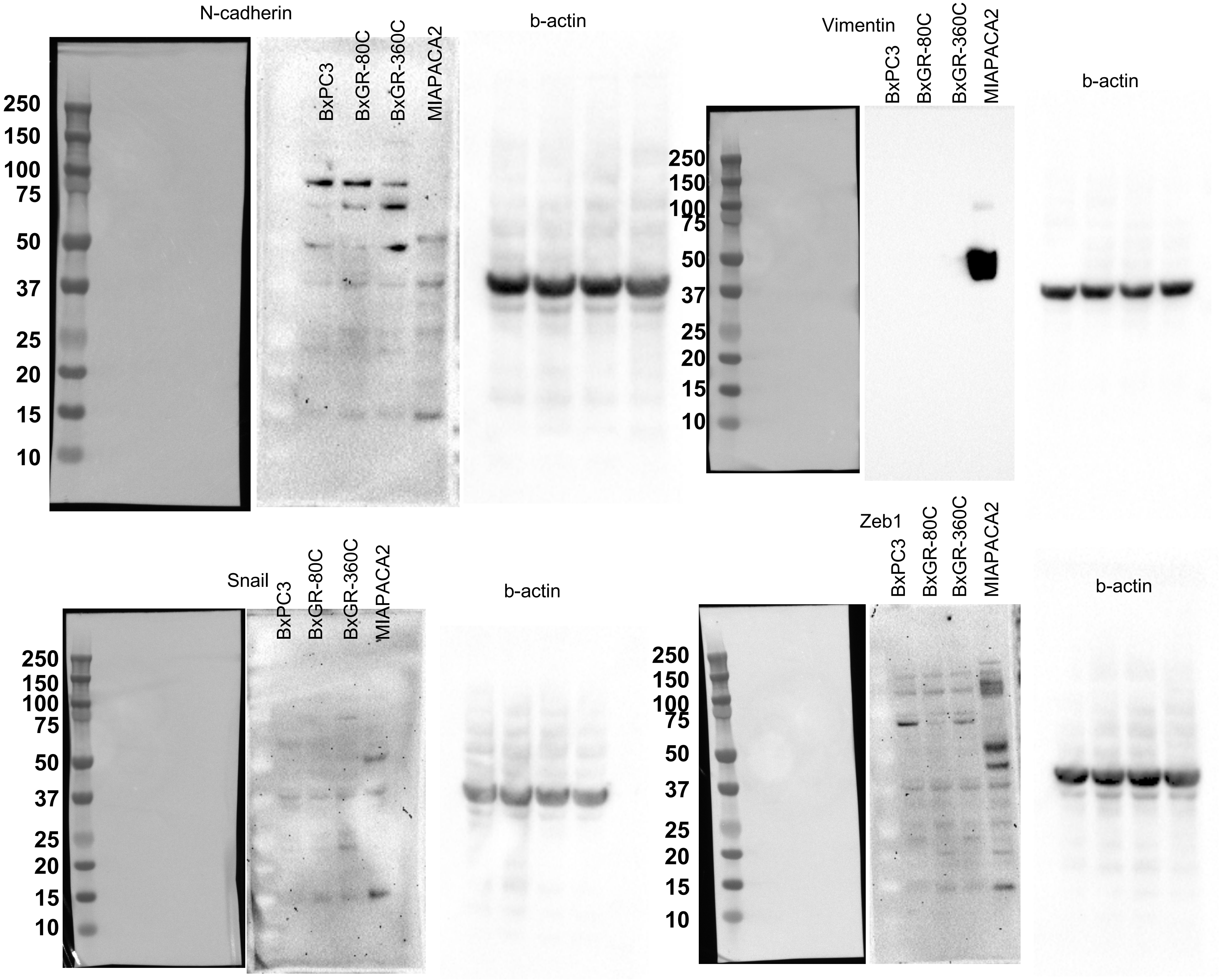

Supporting information. Original western blots of gemcitabine resistant subclones.
